# Standardized Assessment of Biodiversity Trends in Tropical Forest Protected Areas: The End Is Not in Sight

**DOI:** 10.1371/journal.pbio.1002357

**Published:** 2016-01-19

**Authors:** Lydia Beaudrot, Jorge A. Ahumada, Timothy O'Brien, Patricia Alvarez-Loayza, Kelly Boekee, Ahimsa Campos-Arceiz, David Eichberg, Santiago Espinosa, Eric Fegraus, Christine Fletcher, Krisna Gajapersad, Chris Hallam, Johanna Hurtado, Patrick A. Jansen, Amit Kumar, Eileen Larney, Marcela Guimarães Moreira Lima, Colin Mahony, Emanuel H. Martin, Alex McWilliam, Badru Mugerwa, Mireille Ndoundou-Hockemba, Jean Claude Razafimahaimodison, Hugo Romero-Saltos, Francesco Rovero, Julia Salvador, Fernanda Santos, Douglas Sheil, Wilson R. Spironello, Michael R. Willig, Nurul L. Winarni, Alex Zvoleff, Sandy J. Andelman

**Affiliations:** 1 Moore Center for Science, Conservation International, Arlington, Virginia, United States of America; 2 Wildlife Conservation Society, Bronx, New York, United States of America; 3 Center for Tropical Conservation, Duke University, Durham, North Carolina, United States of America; 4 Department of Environmental Sciences, Wageningen University, Wageningen, The Netherlands; 5 School of Geography, Mindset Interdisciplinary Centre for Tropical Environmental Studies, University of Nottingham Malaysia Campus, Selangor, Malaysia; 6 HP Sustainability, HP Inc., Palo Alto, California, United States of America; 7 Escuela de Ciencias Biológicas, Pontificia Universidad Católica del Ecuador, Quito, Ecuador; 8 Forest Research Institute Malaysia, Kepong, Selangor, Malaysia; 9 Conservation International Suriname, Paramaribo, Suriname; 10 Wildlife Conservation Society—Lao PDR Program, Vientiane, Lao PDR; 11 Organization for Tropical Studies, La Selva Biological Station, Puerto Viejo de Sarapiqui, Costa Rica; 12 Center for Tropical Forest Science, Smithsonian Tropical Research Institute, Republic of Panama; 13 Enterprise Services, Hewlett Packard Enterprise, Palo Alto, California, United States of America; 14 Centre ValBio, Ranomafana, Madagascar; 15 Stony Brook University, Stony Brook, New York, United States of America; 16 Universidade Federal do Pará, Museu Paraense Emílio Goeldi, Belém, Pará, Brasil; 17 Hewlett Packard Enterprise Big Data, Palo Alto, California, United States of America; 18 Udzungwa Ecological Monitoring Centre, Udzungwa Mountains National Park, Tanzania; 19 Sokoine University of Agriculture, Morogoro, Tanzania; 20 Institute of Tropical Forest Conservation (ITFC), Mbarara University of Science and Technology (MUST), Mbarara, Uganda; 21 Department of Biology, Western University, London, Ontario, Canada; 22 Wildlife Conservation Research Unit (WildCRU), University of Oxford, Oxford, United Kingdom; 23 Wildlife Conservation Society—Congo Program, Brazzaville, Republic of Congo; 24 Department of Biology, Yachay Tech University, Urcuquí, Imbabura, Ecuador; 25 Tropical Biodiversity, MUSE—Museo delle Scienze, Trento, Italy; 26 Department of Wildlife Ecology and Conservation, University of Florida, Gainesville, Florida, United States of America; 27 Department of Ecology and Natural (INA) Resource Management, Norwegian University of Life Sciences (NMBU), Ås, Norway; 28 Center for International Forestry Research, Bogor, Indonesia; 29 National Institute for Amazonian Research (INPA), Manaus, Amazonas, Brazil; 30 Department of Ecology & Evolutionary Biology and Center for Environmental Sciences & Engineering, University of Connecticut, Storrs, Connecticut, United States of America; 31 Research Center for Climate Change, University of Indonesia, Depok, Indonesia; Princeton University, UNITED STATES

## Abstract

Extinction rates in the Anthropocene are three orders of magnitude higher than background and disproportionately occur in the tropics, home of half the world’s species. Despite global efforts to combat tropical species extinctions, lack of high-quality, objective information on tropical biodiversity has hampered quantitative evaluation of conservation strategies. In particular, the scarcity of population-level monitoring in tropical forests has stymied assessment of biodiversity outcomes, such as the status and trends of animal populations in protected areas. Here, we evaluate occupancy trends for 511 populations of terrestrial mammals and birds, representing 244 species from 15 tropical forest protected areas on three continents. For the first time to our knowledge, we use annual surveys from tropical forests worldwide that employ a standardized camera trapping protocol, and we compute data analytics that correct for imperfect detection. We found that occupancy declined in 22%, increased in 17%, and exhibited no change in 22% of populations during the last 3–8 years, while 39% of populations were detected too infrequently to assess occupancy changes. Despite extensive variability in occupancy trends, these 15 tropical protected areas have not exhibited systematic declines in biodiversity (i.e., occupancy, richness, or evenness) at the community level. Our results differ from reports of widespread biodiversity declines based on aggregated secondary data and expert opinion and suggest less extreme deterioration in tropical forest protected areas. We simultaneously fill an important conservation data gap and demonstrate the value of large-scale monitoring infrastructure and powerful analytics, which can be scaled to incorporate additional sites, ecosystems, and monitoring methods. In an era of catastrophic biodiversity loss, robust indicators produced from standardized monitoring infrastructure are critical to accurately assess population outcomes and identify conservation strategies that can avert biodiversity collapse.

## Abstract 2

In contrast to other reports, inaugural results from a pan-tropical camera trap network suggest that tropical forest protected areas maintain their biodiversity of large and medium ground-dwelling mammals and birds.

## Author Summary

Humans are currently driving numerous animal species toward extinction. Species loss is especially high in tropical regions where most species live and where biodiversity threats are severe. Protected areas such as national parks are the cornerstone of species conservation, but whether protected areas really sustain animal populations and prevent extinction has been debated. This applies particularly to understudied areas such as tropical forests, for which high-quality data are usually not available. We used camera traps to monitor populations of ground-dwelling mammals and birds in 15 protected tropical forests around the world and then used powerful analytics to determine their population trends. We found that some animal populations increased while others decreased. However, the number and distribution of species in these areas did not decline during the 3–8 years we examined. These results are more optimistic about the success of protected areas than were previous studies. Our study demonstrates the importance of standardized monitoring for conservation.

## Introduction

Rates of human population growth are among the highest in the tropics, and natural resource-based economies in many developing countries subject tropical landscapes to rapid rates of land conversion. As a consequence, defaunation—animal loss ranging from local population decline to species extinction [1]—is most extreme in the tropics [1,2], with projected land-use changes expected to cause widespread loss of biodiversity [3]. Tropical mammals and birds, and the primary forests that support them, are essential [4]. Their loss can prompt cascading effects throughout ecosystems [5,6], with “empty forest syndrome” [7] negatively affecting ecological function and human wellbeing [8]. In response to the biodiversity crisis, nearly two hundred nations have committed to increase coverage of effective protected areas, prevent extinctions of threatened species, and improve the status of species in decline by 2020 under the Convention on Biological Diversity (CBD) Aichi Biodiversity Targets.

Two key information challenges inhibit progress on achieving Aichi Targets 11 (Protected Areas) and 12 (Preventing Extinctions). The first challenge is the significant disparity in the amount of tropical biodiversity data that exists in comparison with higher latitude regions [9]. The magnitude of the disparity is compounded by the fact that species richness is highest in the tropics. This data void prevents rigorous evaluation of tropical species responses to threats, which precludes targeted conservation efforts [10,11]. The second challenge is the quality of available biodiversity data in the tropics. In particular, there is a lack of primary in situ data on populations in tropical protected areas [12], which results in conclusions based on aggregated secondary data and expert opinion. For example, the most comprehensive evaluation of the effectiveness of tropical forest protected areas was largely based on expert opinion and suggested that many parks were failing [13]. The gravity of conservation decisions necessitates more objective evaluations using primary data and robust indicators of biodiversity change [14].

**Fig 1:**
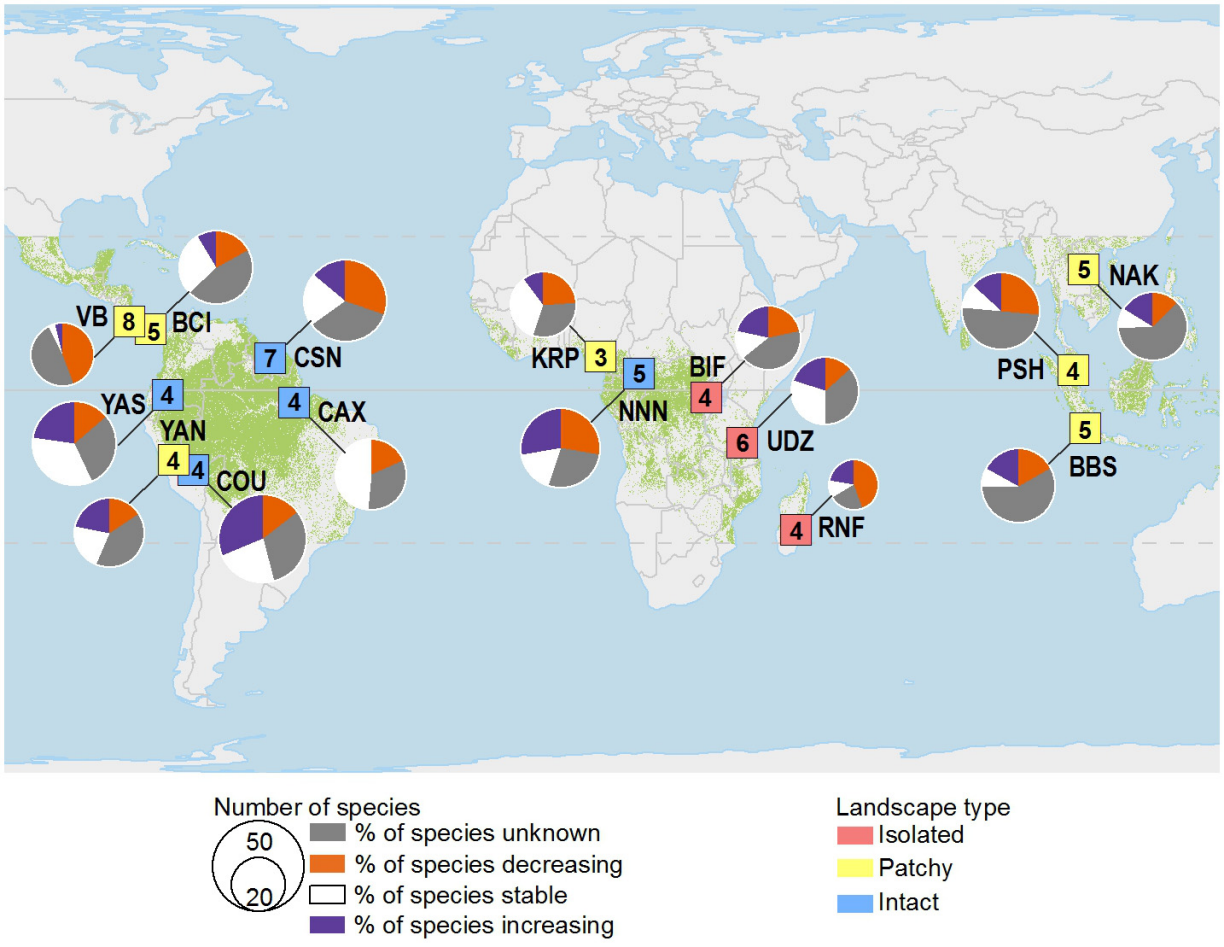
TEAM sites. Trends in occupancy for mammal and bird species in 15 tropical protected areas assessed with standardized surveys using camera traps. The fraction of populations with unknown, decreasing, stable, or increasing occupancy is shown for each site. The type of landscape is indicated by marker color, and the number of years of camera trap data is indicated inside the square marker. Green shading depicts tropical forest. See S1 and S2 Tables for numerical data and information corresponding to 3-letter site codes.

**Fig 2:**
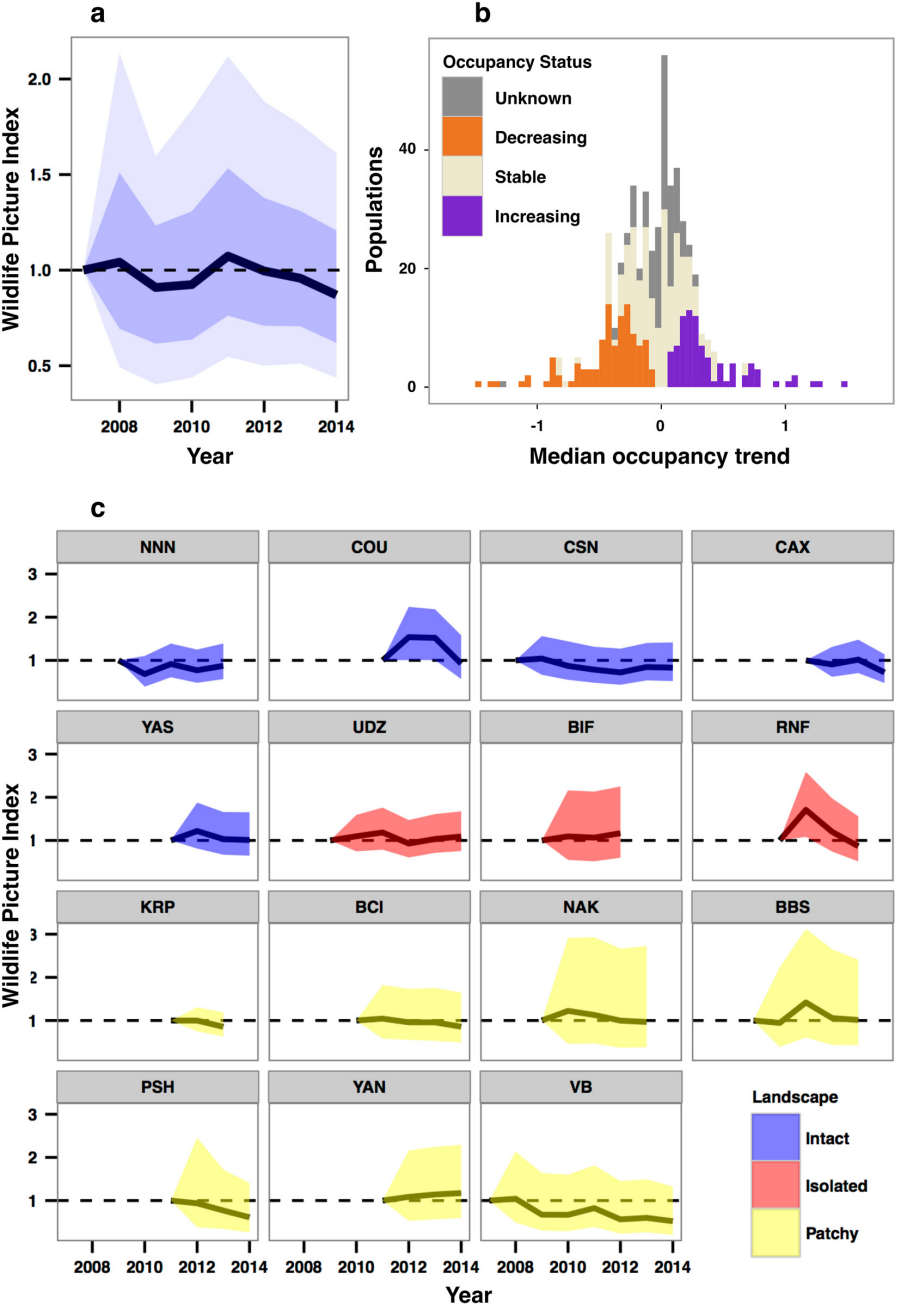
WPI. Overall (a), frequency histogram of occupancy trends and population occupancy status (b), and WPI by site and landscape (c). Shading depicts 50th and 80th (a) or 80th (c) percentile intervals. Labels (c) represent site codes (S1 Table). The WPI Analytics System is accessible at http://wpi.teamnetwork.org. Public access allows anyone to monitor ground-dwelling trends of mammal and bird species in these protected areas. See S4 Table for numerical data for Fig 2A, S2 Table for Fig 2B, and S5 Table for Fig 2C.

Camera traps that photograph animals as they pass by sensors allow for noninvasive surveys in even the most remote places. The Tropical Ecology Assessment and Monitoring Network (TEAM), established in 2002 to help fill the tropical data void, uses large-scale arrays of camera traps to systematically monitor terrestrial (i.e., ground-dwelling) mammals and birds in tropical protected areas on an annual basis (Materials and Methods). TEAM includes 17 sites, 15 of which have three or more years of camera trap data (Fig 1 and S1 Table). Combined, the surveys produce approximately 500,000 images per year and sample over 3,200 km^2^ to inform management of more than 61,000 km^2^ of protected areas. TEAM currently monitors 511 populations from 244 ground-dwelling vertebrate species, which include 41 Threatened (TH), 25 Near Threatened (NT), 169 Least Concern (LC), and nine Data Deficient (DD) species (S2 Table). To evaluate populations via an extensive data collection infrastructure, TEAM uses occupancy analyses that correct for detection probability, as well as the Wildlife Picture Index (WPI), a robust biodiversity indicator developed for camera-trap data and designed to evaluate composite biodiversity trends by being sensitive to changes in species occupancy, richness, and evenness (Methods) (Fig 2).

Occupancy is the estimated probability of a species occurrence at a site [15]. Although the relationship between abundance and occupancy is not linear [16,17], empirical studies have documented a correlation in field settings [18]. Thus, occupancy is an indicator of abundance, and provides information on both abundance and extinction likelihood [19]. Because of the rarity of most tropical mammal and bird species, detections generally are too infrequent to adequately estimate abundance; occupancy provides the best obtainable metric for assessing infrequently detected tropical vertebrates because it requires fewer detections than do metrics of abundance [20].

Here, we assess current occupancy trends of terrestrial mammals and birds in tropical forest protected areas throughout the Neotropics, Africa, and Southeast Asia. Furthermore, we aggregate population-level occupancy trends into composite trends with the WPI to examine the status of tropical vertebrate biodiversity in protected areas at the global scale and at the local level. Based on the properties of the composite index [21,22], we expect decreases in the WPI if (1) overall occupancy declines, but richness and evenness are constant, (2) species evenness declines, but richness and occupancy are constant, or (3) richness declines but occupancy and evenness are constant. In contrast, we expect no trend in the WPI if occupancies of particular species vary while overall occupancy, richness, and evenness do not change significantly.

## Results

During the last 3–8 y, the occupancy of 84 mammal (21.1%) and 27 bird populations (23.9%) exhibited significant declines, the occupancy of 94 mammal (23.6%) and 17 bird populations (15%) was stable, and the occupancy of 70 mammal (17.6%) and 18 bird populations (15.9%) exhibited significant increases. Due to infrequent detection and, therefore, reduced power to detect change (Materials and Methods), the occupancy status of the other 150 mammal (37.7%) and 51 bird populations (45.1%) was classified as unknown (Table 1). Of the 201 unknown status populations, 97 were of species evaluated at other TEAM sites, 48 were of species with consistently unknown occupancy status at multiple sites, and 56 were species found at a single site.

**Table 1:** Detection level and occupancy status summary.

		Number of Populations	
Camera Trap Detection Level	Significantly Decreasing Occupancy	No Detectable Change in Occupancy	Significantly Increasing Occupancy
**High** (Case 1), >8% annual detection	31	48 (Classified as “Stable”)	26
**Medium** (Case 2), <8% annual detection, ≥5 detections per year	32	63 (Classified as “Stable”)	33
**Low** (Case 3), <5 detections per year	48	201 (Classified as “Unknown”)	29

Occupancy trends did not differ significantly between vertebrate classes, the International Union for Conservation of Nature (IUCN) Red List statuses, or body mass categories (S1A–S1C Fig). The proportion of populations with unknown occupancy status differed significantly by dietary guild, landscape connectivity, and hunting level (S1D–S1F Fig; S1 Results). Importantly, while the variance in occupancy trends decreased over time, trends did not differ significantly based on the number of years of camera trap data (S2 Fig). This indicates that, despite differences in time series lengths among sites, the proportion of populations with increasing, decreasing, stable, or unknown occupancy status was consistent over time.

We document that (1) systematic decreases or increases in occupancy, richness, and evenness of mammal and bird populations did not occur in general (Fig 2a); (2) the occupancy dynamics of vertebrate populations were variable (Fig 2B and S3 Fig); (3) protected areas did not evince systemic declines in occupancy, richness, or evenness (Fig 2C); and (4) differences in occupancy dynamics were not significantly related to monitoring duration (S2 Fig). The lack of a significant difference in occupancy trends based on IUCN Red List Status suggests that populations of TH and NT species in these protected areas are faring as well as populations of LC species during the examined time period (S1B Fig).

## Discussion

Using unparalleled, standardized, in situ camera-trap data and robust analytics, we found that 15 protected areas throughout the world’s tropical forests have not exhibited systematic declines in occupancy, richness, or evenness of terrestrial mammal and bird species despite exhibiting extensive variability in population-level occupancy trends during the last 3–8 y. In contrast to reports of declining tropical biodiversity based on expert opinion [13] and other biodiversity indicators [23], our inaugural pantropical assessment suggests less dire outcomes.

Meeting Aichi Targets requires systematic monitoring [24,25] coupled with indicators to track conservation progress [26]. Most published data sources used with Aichi Target 12 indicators are relatively old given the ten-year time frame for evaluating progress under the CBD [26], are biased geographically toward temperate areas [27], rely heavily on expert opinion or disparate sources of information [28], may suffer from publication bias, and do not account for imperfect detection, which can bias estimates [29]. In contrast, the WPI is the first Target 12 indicator that focuses on tropical species, uses current in situ data, and accounts for imperfect detection. The results and approach we present fill an important gap in the CBD indicators for Aichi Target 12 [30] by providing near real-time, essential biodiversity information [14] on species and community characteristics (i.e., occupancy, richness, and evenness) for elusive animals in tropical forests—understudied ecosystems with high concentrations of species.

Our results contrast with severe biodiversity declines estimated by the Living Planet Index (LPI), which is also an Aichi Target 12 indicator [23]. For example, the LPI reports a 56% decline in tropical populations, a 39% decline in global terrestrial populations, and an 18% decline in terrestrial populations in protected areas between 1970 and 2010. Several factors may contribute to different results between the WPI and the LPI, in particular, the (1) temporal duration, (2) exclusion versus inclusion of nonprotected areas, and (3) level of community representativeness.

Assessment of populations for conservation planning requires long-term demographic data, especially for long-lived animals. Although it is possible that 3–8 y is an insufficient duration for detecting meaningful change, power analyses indicate that significant declines in the WPI can be detected within this timeframe [22]. Furthermore, we found no significant differences in occupancy trends over time with respect to the number of years of data collection. Nevertheless, continued monitoring is necessary to distinguish whether the detected occupancy trends reflect short-term fluctuations [31] or false stability.

Although the status of terrestrial vertebrate populations in protected areas may be less dire than previously suggested, we caution that our results are not generalizable to other, nonprotected landscapes, in which many species encounter habitat loss, hunting, and other threats [32–34]. For example, the TEAM site in the Udzungwa Mountains supports much higher species richness than a nearby forest with uncontrolled illegal hunting [35]. Protected areas currently cover approximately 13% of land, providing a critical conservation tool. Nevertheless, maintenance of species occupancy, richness, and evenness within protected areas is likely insufficient to stem the ongoing global extinction crisis, because numerous species exist outside of current protected area coverage, or only a fraction of their geographic ranges are protected [36]. The LPI reports more than twice the rate of decline when terrestrial populations outside of protected areas are considered; we recommend future application of the WPI to nonprotected areas for a comparable global examination.

How well particular species populations represent broader biodiversity trends may also influence differences in the conclusions from the WPI and LPI. Both are composite indices that calculate a geometric mean for the included populations (Materials and Methods). However, the LPI uses population data from disparate sources and geographic locations, while the WPI calculates the geometric mean for all species observed in a community using a standardized sampling protocol. As such, the WPI provides complete community-level representativeness that captures the consequences of community-level processes such as trophic cascades and compensatory dynamics.

Trophic cascades may have occurred at some of the TEAM sites. We found that species with substantial home range requirements, such as large carnivores [37], appear especially affected by habitat loss outside of protected areas (S4 Fig); this is particularly true for small reserves (e.g., Bwindi Impenetrable Forest [BIF], Pasoh Forest Reserve [PSH], S1 Table). Both BIF and PSH have high proportions of herbivores with increasing occupancy and carnivores with decreasing occupancy; declines in carnivore occupancy may have released herbivores from top-down control. In such a situation, variability would occur at the population level, but little change in overall occupancy, richness, or evenness at the community level would be reflected as community stability by the WPI. Similarly, if higher predation from increased carnivore occupancy had caused declines in herbivore occupancy at the Congo site (Nouabali Ndoki [NNN]), community-level stability would also be observed (S4 Fig).

Compensatory dynamics, in which changes in one population are counterbalanced by changes in another population, may have contributed to the community-level stability measured by the WPI. Multiple global studies have documented the maintenance of species richness and other community characteristics despite population-level changes. For example, a meta-analysis of disturbance studies indicated that compensatory dynamics contributed to observed community-level resiliency, that is, the lack of a strong relationship between variability in species-level colonization and extinction events and community-level dynamics [38]. Furthermore, a global analysis of population-level time series illustrated changes in species composition over time, but found no evidence for systematic change in species richness over time, indicating species substitution rather than loss [39].

At the population-level, the occupancy of numerous populations at TEAM sites declined significantly, and even more were detected too infrequently to evaluate. This raises the question of whether defaunation [1] is occurring in these protected areas despite community-level stability. Defaunation may, of course, have occurred in these protected areas prior to the onset of monitoring. In addition, the synergistic effects of human activity and natural demographic variation may have also affected many populations during the examined time period. For example, we found a higher proportion of populations with unknown occupancy status in patchy landscapes. This is consistent with fragmentation disrupting source–sink dynamics and causing increases in the incidence of species rarity. We also found a smaller proportion of hunted populations that were of unknown occupancy status. This is consistent with hunting causing Allee effects that can result in increased local extinction. At the same time, numerous other factors, such as resource limitation, disease, extreme climate events, and environmental stochasticity may have also driven occupancy trends [40]. Because most species are rare and observed infrequently [41], we expect that occupancy trends would not be measurable for some populations. In particular, variation in the proportion of unknown occupancy statuses among guilds is likely due to energetic constraints that result in fewer individuals from species of higher trophic levels [42].

Assessing progress on meeting Aichi Target 11 requires measures of the effectiveness of protected areas [43], but current evaluations (e.g., Protected Area Management Effectiveness Assessments [PAME] [30]) do not include reliable information on such biodiversity outcomes. The data collection infrastructure and analytics that we have leveraged in this study provide a critical solution that is easily transferable to additional sites, ecosystems, and monitoring methods (e.g., acoustic data). Our infrastructure works seamlessly as additional data are included, efficiently manages monitoring data, and computes complex analytics. Importantly, our results are publically available (wpi.teamnetwork.org) so that anyone can monitor population trends in these protected areas. With this approach, we envision a world in which conservation decisions are based on large quantities of standard biodiversity data that are collected globally, shared openly, analyzed rapidly, and synthesized into indicators that assess the effectiveness of protected areas and specific conservation tactics. For the first time, conservation can now be based on near real-time assessment of population trends that are unbiased and sensitive to demographic change. Our highly scalable approach can be readily extended to governments and organizations with vested interests in monitoring wildlife, such as national park networks. Expansion of this new paradigm provides a global opportunity to identify successful conservation strategies to avert biodiversity collapse.

## Materials and Methods

### Data

#### Field data collection

The data presented in this paper come from camera trap images collected by a global tropical forest monitoring network—TEAM. TEAM is a consortium of three core partners—Conservation International, The Wildlife Conservation Society, and The Smithsonian Institution—and over 80 academic and local partners spanning 15 countries and 17 sites in tropical forests around the world. Data were collected between 2007 and 2014 from sites throughout Africa, Asia, and Latin America. We analyzed data from 15 sites, each of which was surveyed for three or more years (Fig 1 and S1 Table). TEAM monitors mammal and bird communities using camera trap arrays following a standardized protocol [44–46]. At each site, 60–90 camera traps are deployed at a density of 1 camera per 1 or 2 km^2^, depending on the forested extent of the site. Each camera trap point is deployed for at least 30 consecutive days during the dry season, which is defined as months with <100 mm average rainfall, or the drier part of the year at sites with no dry season. More details on the field methods can be found in [47].

#### TEAM monitoring sites

Sites were chosen to span moist tropical forest rainfall and seasonality gradients [48], land use change gradients [49], and projected climate change gradients [50]. The sites are distributed in proportion to tropical forest cover on each continent; half of the sites are in South and Central America, roughly one quarter are in Africa, and the rest are in Southeast Asia.

#### Criteria for species inclusion in TEAM monitoring

Mammal and bird species captured on camera trap images were only monitored and reported in this study if (1) body mass was greater than or equal to 100 g [51,52], (2) data [53–55] indicated the species spends a large proportion of its time on or near the ground, or (3) data suggested a species was arboreal and there was at least one TEAM site at which the species was detected in five or more photographic events (collection of images separated by at least 1 min) for every year that data were collected. A complete list of all populations and species with relevant metadata is in S2 Table.

#### Data preparation

Observational records were condensed into presence–absence matrices, one for each site, species, and year, where the rows corresponded to sampling points and the columns correspond to time periods (days). For each sampling point and day, the cells in these matrices were occupied by a “1” if the species was photographed, a “0” if the species was not photographed, or “NA” if the point was not actively sampled on this day. To reduce model computation time and increase efficiency, we grouped observations into 15 time periods for each site, species, and year so that each time period equaled approximately 7–8 d of sampling. After grouping, observations remained as 0s and 1s. Grouping does not affect model estimates, but only the units of the estimated detection probabilities for each species [15]. All data processing was conducted with scripts programmed in the language R [56].

### Occupancy Modeling

We first examined how well dynamic occupancy models recovered dynamics based on the number of detections. Specifically, we explored different numbers of detections and evaluated how well models recovered parameters. For example, for species with less than five detections per year, models always resulted in occupancy estimates close to one, as the models were unable to distinguish between low detection and low occupancy. From these exploratory analyses, we identified three cases based on the species detection level per site. We thereafter used three different models depending on detection level. For populations with annual detection greater than 8% (Case 1), we used a Bayesian dynamic occupancy model that accounted for imperfect detection and employed covariates [57]. For populations with five or more detections per year but annual detection less than 8% (Case 2), we used the same Bayesian dynamic occupancy model as in Case 1, but omitted covariates. For populations with fewer than five detections per year (Case 3), we calculated the naïve (i.e., observed) occupancy of a population. Hereafter, we describe each of these three approaches in detail.

#### High detection (Case 1)

Common populations (N = 105 populations). For populations that had an observed average annual detection (number of times species *i* was detected across all sampling points *j* and time periods *k* averaged over *t* years (∑*y*_*ijkt*_/*t* ∑*j*_*t*_*k*_*t*_) that was greater than 8%, we used a hierarchical dynamic occupancy modeling approach with covariates [57]. Here, the ecological process that influences occupancy is modeled separately from the observation process (i.e., detection), which is considered imperfect. In the ecological process model, the incidence of a species *i* in the first year of observation is an unobserved latent variable *Z*_*i1*_, resulting from a Bernoulli process with expected probability ψ_*i1*_: *Z*_*ij1*_ ~ Bernoulli(ψ_*i1*_). For subsequent years (year > 1), the site remains occupied by the species with probability *ɸ*_*i*_, or goes locally extinct from that site with probability (1−*ɸ*_*i*_), where *ɸ*_*i*_ is the apparent survival of the species from one year to the next and (1−*ɸ*_*i*_) is the local extinction probability. If the species did not occupy the site in year *t* (*Z*_*it*_ = 0), it can colonize this site by the following year (*t +1)* with probability *γ*_*i*_ [57]. The dynamics of a species *i* can be described recursively for any two contiguous years as:

$$\psi_{i\left(t+1\right)}=P\left(Z_{i\left(t+1\right)}=1\right)=\;Z_{it}\phi_{i}+\;\left[1-\;Z_{it}\right]\gamma_{i}$$ (1)

The observation process of the model assumes the observations for each species *i* at each sampling point *j*, year *t*, and observation period *k*, *y*_*ijtk*_, as realizations of a Bernoulli process with mean *Z*_*it*_ *p*_*i*_, where *p*_*i*_ is the detection probability of species *i* at year *t*: *y*_*ijtk*_ *~* Bernoulli(*Z*_*it*_ *p*_*i*_). This takes into account imperfect detection at the sampling point (i.e., false negatives) and provides an unbiased estimate of occupancy. Under this model, closure is assumed within years but not between years; thus, colonization and extinction only occur on an annual basis. Covariates were selected to test the effects of climate (i.e., rain, temperature) and anthropogenic factors (i.e., people, edges) on occupancy, survival, and colonization. The hypotheses, stated as equations, describe the relationship between state variables and covariates:

For first year occupancy *ψ*_*ij1*_:

$$\text{logit}\left(\psi_{ij1}\right)=\;\alpha +\;\beta_{1}*elev_{j}+\;\beta_{2}*peop_{j1}+\;\beta_{3}*edge_{j}.$$ (2)

For survival probability *ɸ*_*ijt*_:

$$\text{logit}\left(\phi_{ijt}\right)=\;\alpha_{\phi }+\;\beta_{4}*rain_{t}+\;\beta_{5}*maxTemp_{t}+\;\beta_{6}*minTemp_{t}+\;\beta_{7}*peop_{jt},$$ (3)

and colonization *γ*_*ijt*_:

$$\text{logit}\left(\gamma_{ijt}\right)=\;\alpha_{\gamma }+\;\beta_{8}*rain_{t}+\;\beta_{9}*maxTemp_{t}+\;\beta_{10}*minTemp_{t}+\;\beta_{11}*peop_{jt}.$$ (4)

We used three types of covariates for each site: sampling point covariates (*elev*, *edge*), time-dependent covariates (*rain*, *maxTemp*, *minTemp*), and a covariate that was sampling-point- and time-dependent (*peop*).

Sampling point covariates were camera trap, elevation above sea level (*elev*), and camera trap distance to the nearest edge (*edge*) that could be a road, river, forest, protected area boundary, or settlement. Each camera trap location was determined in the field using a handheld GPS device. Elevation (meters above sea level) of each camera trap was obtained from a 90 m spatial resolution void-filled Shuttle Radar Topography Mission (SRTM) digital elevation model from the Consortium for Spatial Information [58]. Distance to edge was calculated using a geoprocessing tool in ArcGIS that determines the distance from each feature in the “input features” to the nearest feature in the “near features.” The camera trap points were the “input features,” and a polygon or polyline shapefile of the edge type (e.g., roads, rivers) were the “near features.” Local data source shapefiles were used for each edge type. When a local spatial data layer was not available for a particular edge type, we used the Global Roads Open Access Data Set, Version 1 [59], forest classes from Globcover V2.3 [60], and the World Database on Protected Areas protected area boundaries [61].

Time-dependent covariates changed only between sampling years but not between sampling points. These included cumulative rainfall (mm) during the 6 mo prior to the start of the camera trap deployment (*rain*), average maximum daily temperature (°C) at the site during the 6 months prior to the start of the camera trap deployment (*maxTemp*), and average minimum daily temperature (°C) at the site during the 6 mo prior to the start of the camera trap deployment (*minTemp*). Climate data were extracted from local TEAM climate stations installed at each site that follow a standard protocol for data collection [45]. Rainfall data were aggregated from five-minute resolution measurements and summed over six months. Maximum and minimum temperatures were calculated daily and averaged over six months.

The sampling-point- and time-dependent covariate was the presence or absence of people, as detected by the camera trap at that point in a particular year (*peop*). Presence or absence of people for each site and each year was derived directly from camera trap data at that site; local site managers identified people as another species captured in the images. The people covariate includes all people regardless of their potential benefit or threat to wildlife (e.g., tourists, park visitors, poachers, field personnel, park patrol). All covariate values (except presence or absence of people) were normalized by subtracting the site mean and dividing by the site standard deviation to allow for easier interpretation of effects and to help with model convergence.

We fit the models using Markov chain Monte Carlo (MCMC) methods and the software JAGS [62,63], running through package R2jags in R [64]. Uniformative priors were used for all state variables, latent variables, and covariate parameters (S1 Text). To identify potentially important covariates in the model, we used an indicator variable selection approach where an indicator variable (*w*_*i*_) multiplies each model term *i* (*w*_i_*β_i_*cov_i_) and is given a prior of 0.5 from a Bernoulli distribution (*w*_*i*_ *~* Bernoulli (0.5)) [65,66]. As the MCMC chain runs, the value of *w*_*i*_ reflects the weight of that covariate in the model, or the probability that the variable should be included in the model. If the covariate is very important, E [*w*_*i*_] is close to 1; if the covariate does not have any influence in model performance, E [*w*_*i*_] is close to 0. By running long MCMC chains relative to the number of potential model subsets (for this case 2^11^ = 2,048), it is ensured that most potential models are visited. We used three MCMC chains with 250,000 iterations each, and examined the last 1,000 iterations. At the end of the process, the posterior distribution of the effects (β_i_), where *w*_*i*_ > 0.5, were extracted, and 95% highest posterior density (HPD) were calculated. However, the posterior distribution of occupancy (or any of the other state variables) was estimated from the last 1,000 iterations of the chain sampling the entire model ensemble, representing a model average set over all possible models [16]. The approach is similar to stochastic variable selection [63] with the difference being that the indicator variable does not come from a normal mixture process.

#### Medium detection (Case 2)

Populations with five or more detections per year but less than 8% annual detection (N = 128 populations). For populations that were detected at a site on five or more occasions per year, but that had an annual average observed proportional detection less than 8%, we used the dynamic occupancy modeling approach described above, omitting all covariates. We fitted models using MCMC methods following the approach in [57], using software JAGS [62,63] running through package R2jags in R [64]. JAGS models were based on existing code [57] and were run with three chains of 30,000 iterations each, a thinning rate of three (every third iteration discarded), and a burn-in rate of 29,000 iterations (ignored for calculation of posterior density distributions). Models were checked for convergence by ensuring that the Gelman-Rubin statistic for each parameter was below 1.03 [67]. Model parameters were recovered using the mode of the distribution because many of the posterior distributions were highly skewed. HPD intervals were extracted from the posterior distributions at a 95% level for inference purposes.

#### Low detection (Case 3)

Populations with fewer than five detections per year (N = 278 populations). For populations *i* that were detected an average of fewer than five camera trap points *j* per year *t* at a given site, we calculated the observed “naïve” occupancy (number of points *j* at which a population was detected at least once [*Z*_*ijt*_ *=* 1/number of points sampled]). The error associated with the naïve occupancy was estimated through a Bayesian approach using a beta prior for the occupancy on a binomial distribution [68]. A random sample of 1,000 realizations was drawn from the posterior, and 95% HPD intervals were calculated. Calculations were performed in R using package binom [69].

### Wildlife Picture Index

We have developed cyberinfrastructure for processing and curating data from camera traps through technology partners Hewlett Packard Enterprise, formerly Hewlett Packard Company, and the San Diego Super Computer Center at the University of California, San Diego [70]. The Hewlett Packard Earth Insights program [71] has enabled TEAM to scale up data analyses to monitor near real-time changes in biodiversity. Together, we have implemented the WPI in a near real-time, online analytics system and visual dashboard accessible at http://wpi.teamnetwork.org.

#### WPI calculation

We use the WPI [20] to assess changes in terrestrial vertebrate biodiversity. The WPI is an indicator specifically developed for camera-trap data, designed for use with the TEAM survey, and created to meet the requirements of an indicator for the CBD. Specifically, the WPI tracks changes over time [21] and is sensitive to variation in species richness, species evenness, and changes in species occupancy or abundance. The WPI_*s*,*t*_ is the geometric mean of the occupancies of all the species (*i…n*) in the community *s* scaled by the baseline occupancy for each in year *t* [15]:

$$WPI_{s,t}=\text{exp}\left\{\frac{1}{n}\overset{n}{\underset{i=1}{\sum }}log\frac{\psi_{ist}}{\psi_{is1}}\right\}$$ (5)

This index is unique, because the underlying occupancy models account for variability in detection [72,73] within and among species using unbiased population trends.

The WPI is an ideal metric for evaluating changes in biodiversity because it is sensitive to changes in richness, relative abundance or occupancy, dominance, and other measures of community diversity [21]. We used the approach proposed by Ahumada et al. [74], which allowed us to compute the WPI as a derived quantity from the posterior occupancy distributions (geometric mean of the relative occupancies at each model iteration after model burn-in), resulting in a full posterior distribution for the WPI each year. From these distributions, we extracted the 95% HPD intervals and used the median or mode (whichever was lowest) as the measure of central tendency of the distribution.

The WPI can be aggregated based on spatial extent or species attributes. For example, it can be calculated at the level of a TEAM site (local), of sites on the same continent (regional), and of all sites (global), or for species grouped according to IUCN conservation status or dietary guilds. When aggregating the WPI for sites that started in different years, the WPI distributions are convolved yearly in one distribution, ignoring the baseline of the site that started later in time, because the WPI in the baseline year for a site is always 1 with no error distribution.

#### WPI Analytics System

The WPI Analytics System uses the HPE Vertica Analytic Platform. The system integrates existing data capture systems for TEAM camera trap and covariate data. Any new data or changes to the data are automatically migrated from the TEAM Network’s database to an HPE Vertica system using a versioning scheme to maintain unique versions for each migrated data set, thereby maintaining data integrity. After each migration, the WPI system autotriggers a series of computations for new occupancy values of each species for migrated site data. The compute logic is implemented as HPE Vertica User Defined Transform Functions (UDTF), invoked by a set of dedicated compute thread pools in the WPI application. The Compute Engine leverages HPE Vertica-R interoperability and JAGS library for running MCMC simulations [64]. The WPI system provides complete traceability of input data and simulation results for each run. The system provides alerts and feedback on the progress of compute-intensive simulation jobs during compute/recompute and publish/unpublish tasks in the WPI workflow.

The system model implemented on HPE Vertica runs a large number of MCMC simulations on multiple species—more than 33 million iterations for the existing TEAM data from 15 sites. The last 1,000 iterations for each species at a site per year, yielding approximately 2 million data rows, are used for multilevel WPI computation and analysis. The WPI system is capable of processing much larger volumes of data, running hundreds of millions of iterations and enabling TEAM and others to add new sites and new data sources.

### Occupancy Status and Potential Explanatory Variables

#### Occupancy status categorization

The dynamic occupancy models were mechanistic and captured year-to-year variation in occupancy due to changes in survival and colonization probabilities. To estimate long-term occupancy trends in these populations, we removed temporal variation in the annual occupancy estimates by fitting logistic regression models. Specifically, we fit logistic regression models to the detection-corrected yearly occupancy estimate distributions (Cases 1 and 2) and the raw occupancy distributions (Case 3) for each population *i* as a function of time *t*:

$$\psi_{i}\left(t\right)={\left(1+e^{-\left(\alpha_{i}+\beta_{i}t\right)}\right)}^{-1}$$ (6)

A separate logistic regression through time was fitted for each modeled realization (1,000 realizations for each species population at a site), resulting in a distribution of slopes and intercepts for each population at each site. The median and two-tailed 80th percentile intervals were calculated to determine whether the slopes were significant at the 80th percentile (Fig 2B and S3 Fig). Logistic regressions were fitted in R using the glm function in package stats [56].

Populations were considered to be significantly declining, increasing, or stable based on whether the 80th percentile interval of the slope distribution was entirely below zero, entirely above zero, or neither, respectively (but see below for “unknown” category). In the context of conservation, false positives (i.e., species categorized as declining when actually stable) are preferable to false negatives (i.e., species categorized as stable when actually declining). We therefore used a conservative cutoff (80% credible intervals) for classifying occupancy trends, because it is better to provide an earlier warning signal of occupancy declines that can prompt conservation action [75].

We used log-likelihood goodness of fit tests (G-tests) to assess whether the proportion of populations with increasing, decreasing, or stable occupancy (i.e., demographic status) was independent of case. A significant result indicates significantly different frequency distributions of demographic status among cases [76]. There were no significant differences in the proportion of populations with decreasing, stable, or increasing occupancy between Cases 1 and 2 (i.e., high and medium detection; G-test, G = 0.61, df = 2, *p* = 0.738), but there were significant differences in occupancy for populations modeled as Case 3 (i.e., low detection) (G-test, G = 35.44, df = 4, *p* < 0.001) with a higher proportion of populations classified as stable. This is likely due to reduced ability to detect changes because of very few observations (i.e., fewer than five detections annually) rather than to actual occupancy stability. For this reason, and the potentially negative conservation repercussions from failing to detect change, we classified the occupancy status of all Case 3 “stable” populations as “unknown.”

#### Analysis of occupancy status

We used log-likelihood goodness of fit tests [76,77]) to examine variation in the proportion of populations with increasing, decreasing, unknown, or stable occupancy based on six categorical variables (i.e., class, IUCN status, body size, guild, landscape type, hunting; see below). We accounted for multiple comparisons and controlled for family-wise error rate by applying a Bonferonni correction (α = 0.05/6 = 0.008).

We created S1 and S4 Figs with relative multiple bar (rmb) plots using the R package extracat [78]. Relative multiple bar plots display the relative frequencies of a response variable (i.e., occupancy status) for each combination of explanatory variables (i.e., class, IUCN status, body mass category, guild, landscape type, hunting status). The total sample is divided over a grid-like graphical display, which illustrates the corresponding sample weights for each variable combination.

We used generalized linear models with a binomial distribution to model the proportion of populations per site as a function of continuous predictors (S1 Table). We examined the proportion of populations with (A) increasing or (B) decreasing occupancy within each site using each of the following continuous variables in a separate model: (1) log of protected area size, (2) human population density, (3) proportion forested, (4) edge density, (5) proportion of hunted populations, (6) proportion of not hunted populations, and (7) years of camera trap data. For example: glm(Site Increasing Proportion ~ Site Human Population Density, family = binomial). Pairwise relationships between these continuous variables are shown in S5 Fig.

#### Potential explanatory variables

**(1) Body size and guild.** We used species-level body mass (g) and guild data compiled from published sources [51–55]. Body mass ranged from 107 g to 39,400 kg (S2 Table), with a median of 3,250 g. We evaluated three body mass categories, defined by orders of magnitude as 100–1,000 g (39 bird and 25 mammal species), 1,001–10,000 g (21 bird and 94 mammal species) and >10,000 g (65 mammal species). Each species was categorized as one of four dietary guilds: carnivore (49 mammal species), herbivore (eight bird and 59 mammal species), insectivore (19 mammal species), or omnivore (52 bird and 57 mammal species) (S2 Table).

**(2) Structural landscape connectivity in the zone of interaction (ZOI).** Processes outside of the protected area boundaries may affect mammal and bird populations within the camera trap sampling area. We therefore examined the broader landscapes outside of the protected area boundaries of each TEAM site using the zone of interaction (ZOI) to define spatial extent. The ZOI is the area that has the potential to strongly influence biodiversity at the site based on systematic quantification of surrounding watersheds, migration corridors, and human settlements [32].

To map forest cover at each TEAM site, we use the Global Forest Change (GFC) product [79]. After conducting a sensitivity analysis across a range of thresholds, we applied a 75% threshold to the 2000 forest cover layer in the GFC product, taking into account the gain and loss layers in the product to calculate a forest–nonforest map for each site for 2012. We verified the accuracy of these maps through comparison with independently derived land cover classifications. We then filtered the data to set a minimum patch size of 990 m^2^ (S6A Fig). We calculated two measures of landscape connectivity (edge density and the proportion of forested landscape [80]) from the forest cover data for each TEAM ZOI using the “ClassStat” function in the SDMTools library in R [81]. We square root transformed edge density to normalize its distribution and calculated a pairwise distance matrix using a Mahalanobis distance to remove any correlation between the connectivity metrics [82]. We then ran an unweighted pair group method with arithmetic mean (UPGMA) cluster analysis on the distance matrix and identified the landscape types based on the three clusters produced at a height cutoff of 1.5 in the resulting dendrogram [83] (S6B Fig). Based on visual inspection of forest cover (S6A Fig), we classified the three clusters as: (1) intact landscapes in which the protected areas were indistinguishable from surrounding forest (i.e., CAX, COU, NNN, CSN, and YAS), (2) isolated landscapes in which the protected areas were surrounded by non-forested landscape (i.e., BIF, RNF, and UDZ), and (3) patchy landscapes in which the protected area was embedded in a patchwork of forested and nonforested areas (i.e., KRP, PSH, VB, BBS, YAN, BCI, and NAK).

The dichotomy between forest and nonforest cover in our analyses does not differentiate between natural and anthropogenic land uses. For example, some forested areas may be agricultural landscapes with closed canopies (e.g., palm oil surrounding PSH in Malaysia; banana plantations near VB in Costa Rica) that do not provide benefits to wildlife comparable to native forest [84]. Additionally, some nonforested area may represent natural vegetation that arises from breaks in forest cover (e.g., high elevation areas above tree line at YAN in Peru).

**(3) Protected area size.** To estimate protected area size, we extracted the polygon of the protected area for each TEAM site from the World Database on Protected Areas (WDPA) [85] and verified each polygon with the appropriate local site manager. We calculated the area of each protected area in hectares after reprojecting the polygons to the appropriate local (UTM) coordinate system.

**(4) Hunting.** Field managers at each TEAM site were surveyed about hunting within the protected area where the core TEAM camera trap sampling occurs. We distributed species lists to managers and asked them to mark whether each species at their site list was hunted (yes), not hunted (no), or whether they did not know (unknown). Our measure of hunting was simply the proportion of populations reported as hunted, which relies on expert opinion and does not take the level of hunting pressure into account. Primary data on hunting warrant further investigation.

## Supporting Information

S1 Fig Population occupancy status. By class (a), IUCN category (b), body mass (c), guild (d), landscape type (e), and hunting status (f). Bar height illustrates the relative frequencies of each occupancy status, and bar width illustrates the sample size. Asterisks indicate a significantly different occupancy status. See S2 Table for numerical data.
(TIF)

S2 Fig Population occupancy status and monitoring duration. Frequency histogram of population trends based on the number of years of camera trap data (3–4 y [*n =* 8 sites, 270 populations], 5 y [*n* = 4 sites, 141 populations], or 6 y or more [*n* = 3 sites, 100 populations]). While the variance in occupancy trends decreased with additional years of data, the proportion of populations with increasing (purple), decreasing (orange), stable (white), or unknown (gray) occupancy did not vary significantly based on monitoring duration (G-test, G = 11.36, df = 6, *p* = 0.079, *n* = 511). See S2 Table for numerical data.
(TIF)

S3 Fig Population occupancy over time. Each line depicts the trend of a particular species population monitored by TEAM. Color depicts significantly decreasing (orange), significantly increasing (purple), unknown (gray), or stable (black) occupancy trends. See S2 Table for numerical data.
(TIF)

S4 Fig Occupancy status by TEAM site and guild. The proportion of populations with decreasing (orange), increasing (purple), unknown (gray), or stable (white) occupancy for each guild at each site; *n* = 511 populations. See S1 Table for site information corresponding to the three-letter site codes. Bar length illustrates the proportion of each occupancy status, and bar width illustrates the sample size for each guild at each site. See S2 Table for numerical data.
(TIF)

S5 Fig Pairwise relationships between continuous site-level variables. Upper triangular portion of the matrix contains Pearson correlation coefficients, with the font size proportional to the correlation coefficient. Lower triangular portions of the matrix contain pairwise scatter plots to illustrate associations. “PAsize” is the log of the size of the protected area in hectares. “HuntY” is the proportion of populations at a site that was reported as hunted. “HuntN” is the proportion of populations at a site that was reported as not hunted. “Forested” is the proportion of the ZOI that was forested. “Edges” is the edge density of the ZOI, and “Pop_den” is the human population density in the ZOI. See S1 Table for numerical data.
(TIF)

S6 Fig Forest cover and landscape classification. (a) Green represents forest cover and brown represents non-forested cover for the ZOI of each protected area (Materials and Methods). Bold text is the code for each TEAM site. *x*- and *y*-axis labels are degrees latitude and longitude, respectively. (b) The UPGMA cluster analysis was based on two measures of landscape connectivity: proportion of forested landscape and edge density. The cluster dendrogram depicts three clusters of similar landscapes based on a height (i.e., cluster agglomeration value) of 1.5. See S1 Table for numerical data and corresponding site information.
(TIF)

S7 Fig Important dynamic occupancy covariates. Covariates of initial occupancy (psi), colonization (lambda), and survival (phi) parameters for populations modeled as Case 1. Colors depict coefficient values of covariates identified as important using indicator variable selection (Materials and Methods). Gray shading represents covariates that were not identified as important. The y-axis labels populations by species name and site code. See S1 and S2 Tables for corresponding site and species information. See S6 Table for numerical data.
(TIF)

S1 Results Supplementary results. (DOCX)

S1 Table Summary information for TEAM sites. Including the full site name, site code, country, number of years of camera trap data, number of populations monitored by TEAM, percent of populations for each occupancy status, percent of monitored populations that were reported as hunted or not hunted, landscape connectivity, camera trap sampling area, protected area size, area of the ZOI, human population density (per ha) in the ZOI, and two measures of structural connectivity in the ZOI (proportion forested and edge density).
(PDF)

S2 Table Monitored populations and associated attribute data. The following attributes are listed for each population: Class, Order, Family, Species, IUCN Red List Status (TH = Threatened, NT = Near Threatened, VU = Vulnerable, LC = Least Concern, DD = Data Deficient), Body Mass, Guild (Omnivore, Carnivore, Herbivore, Insectivore), Occupancy Coefficient Slope, Occupancy Status (Increasing, Decreasing, Stable, Unknown), Hunting Status (Hunted: Yes, No, Unknown), Occupancy Model Case (1, 2, 3) and TEAM site code.
(XLSX)

S3 Table AIC comparison of univariate logistic regression models. For (a) the proportion of decreasing occupancy status populations per site and (b) the proportion of increasing occupancy status populations per site. For both decreasing and increasing occupancy status proportions, the null models with no covariates performed better (delta AIC > 2) than all other models.
(PDF)

S4 Table Numerical data for Fig 2A. (CSV)

S5 Table Numerical data for Fig 2C. (CSV)

S6 Table Numerical data for S7 Fig. (CSV)

S1 Text R code. (PDF)

## Abbreviations

BBS: Bukit Barisan
BCI: Barro Colorado Nature Monument-Soberania National Park
BIF: Bwindi Impenetrable Forest
CAX: Caxiuana
CBD: Convention on Biological Diversity
COU: Cocha Cashu-Manu National Park
CSN: Central Suriname Nature Reserve
DD: Data Deficient
GFC: Global Forest Change
HPE: Hewlett Packard Enterprise
HPD: highest posterior density
IUCN: International Union for the Conservation of Nature
KRP: Korup National Park
LC: Least Concern
LPI: Living Planet Index
MCMC: Markov chain Monte Carlo
NAK: Nam Kading
NNN: Nouabali Ndoki
NT: Near Threatened
PAME: Protected Area Management Effectiveness Assessments
PSH: Pasoh Forest Reserve
rmb: relative multiple bar
RNF: Ranomafana
SRTM: Shuttle Radar Topography Mission
TEAM: Tropical Ecology Assessment and Monitoring Network
TH: threatened
UDTF: User Defined Transform Function
UDZ: Udzungwa
VB: Volcan Barva
WDPA: World Database on Protected Areas
WPI: Wildlife Picture Index
YAN: Yanachaga Chimillen National Park
YAS: Yasuni
ZOI: zone of interaction
