# Supplementary figures and images for: Standardized Assessment of Biodiversity Trends in Tropical Forest Protected Areas: The End Is Not in Sight
Source: PLoS Biol. 2016 Jan 19;14(1):e1002357. doi: 10.1371/journal.pbio.1002357 (PMC4718630; doi:10.1371/journal.pbio.1002357)

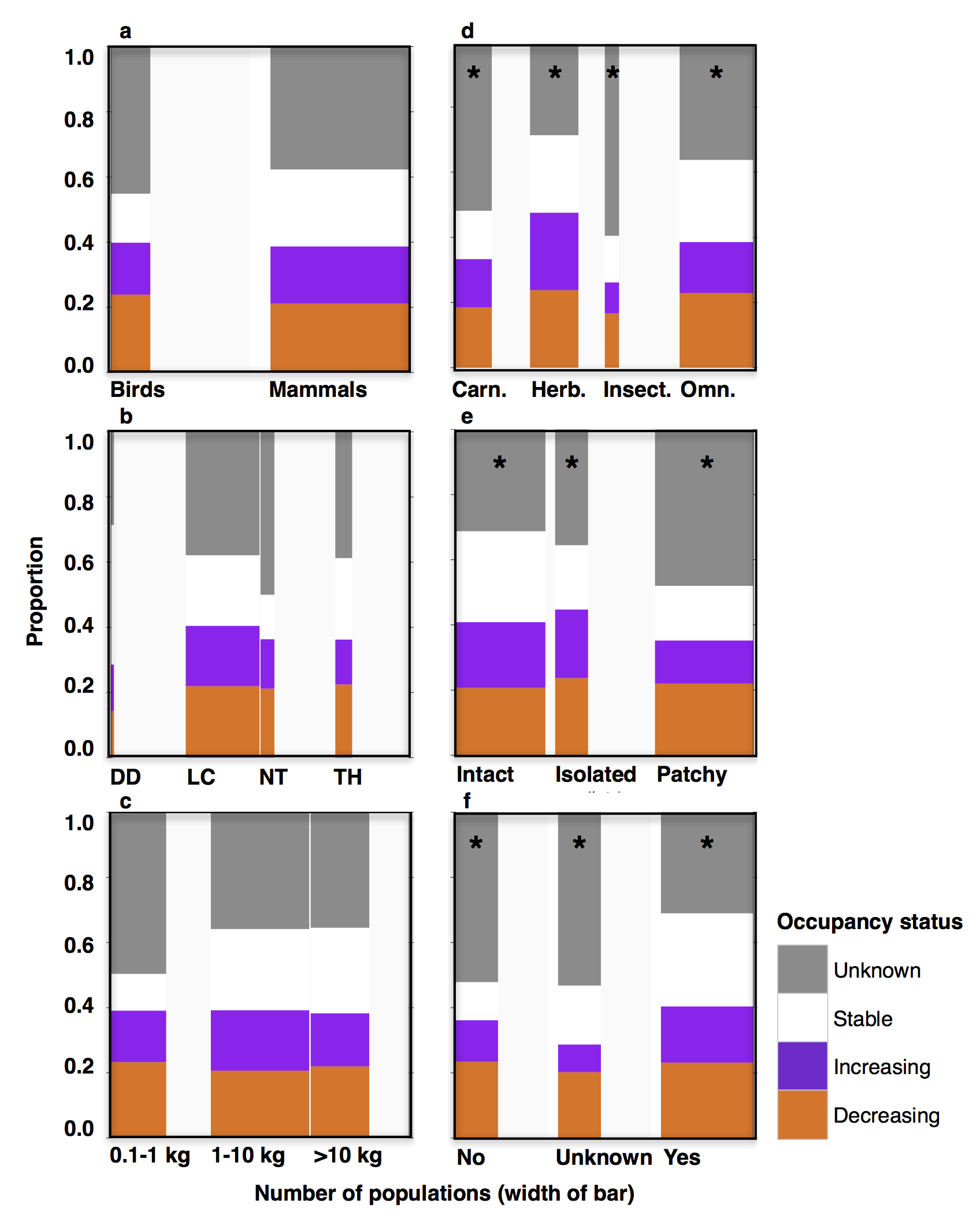

Supplement: S1 Fig — By class (a), IUCN category (b), body mass (c), guild (d), landscape type (e), and hunting status (f). Bar height illustrates the relative frequencies of each occupancy status, and bar width illustrates the sample size. Asterisks indicate a significantly different occupancy status. See S2 Table for numerical data. (TIF) [file pbio.1002357.s001.tif]

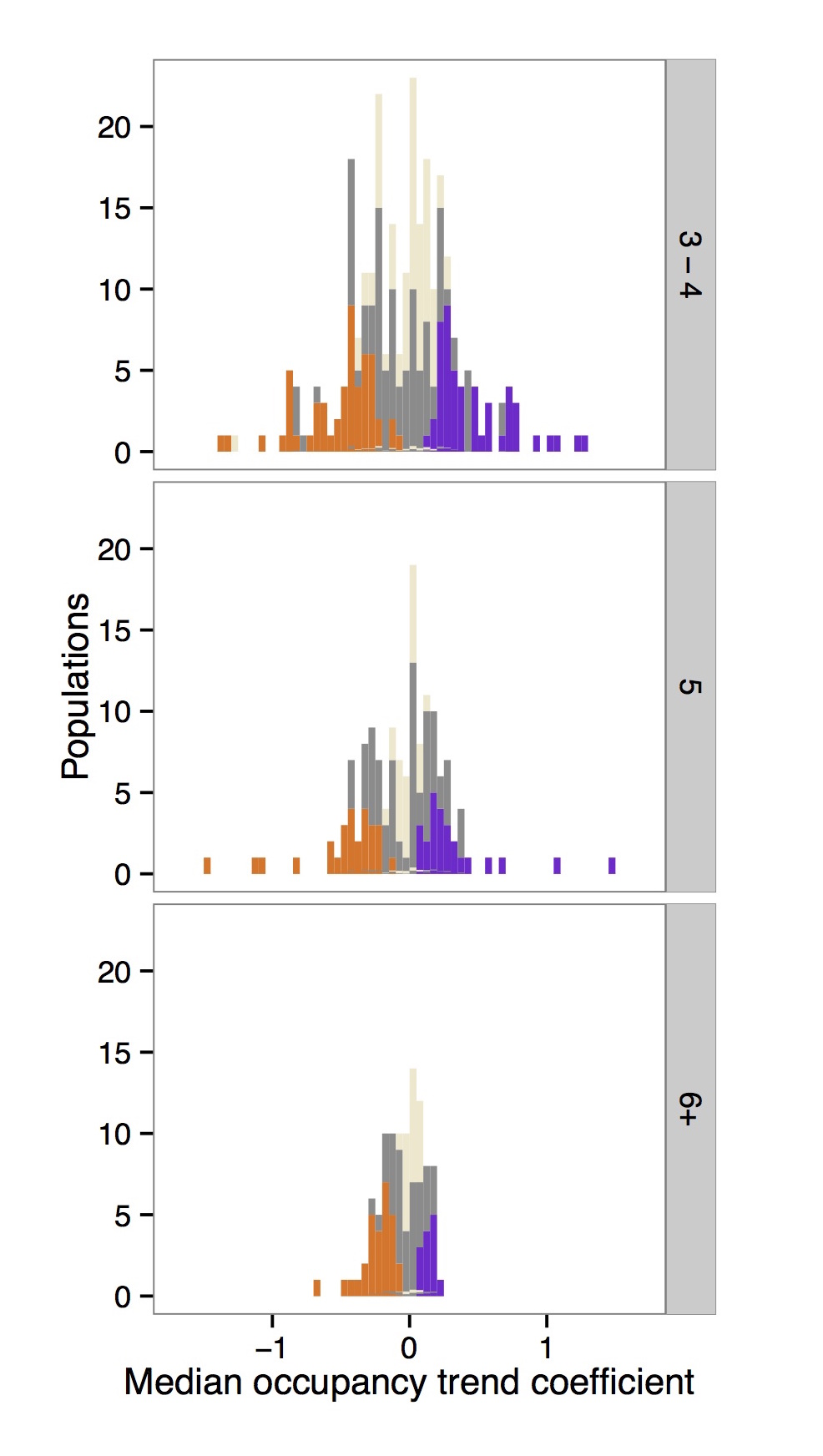

Supplement: S2 Fig — Frequency histogram of population trends based on the number of years of camera trap data (3–4 y [n = 8 sites, 270 populations], 5 y [n = 4 sites, 141 populations], or 6 y or more [n = 3 sites, 100 populations]). While the variance in occupancy trends decreased with additional years of data, the proportion of populations with increasing (purple), decreasing (orange), stable (white), or unknown (gray) occupancy did not vary significantly based on monitoring duration (G-test, G = 11.36, df = 6, p = 0.079, n = 511). See S2 Table for numerical data. (TIF) [file pbio.1002357.s002.tif]

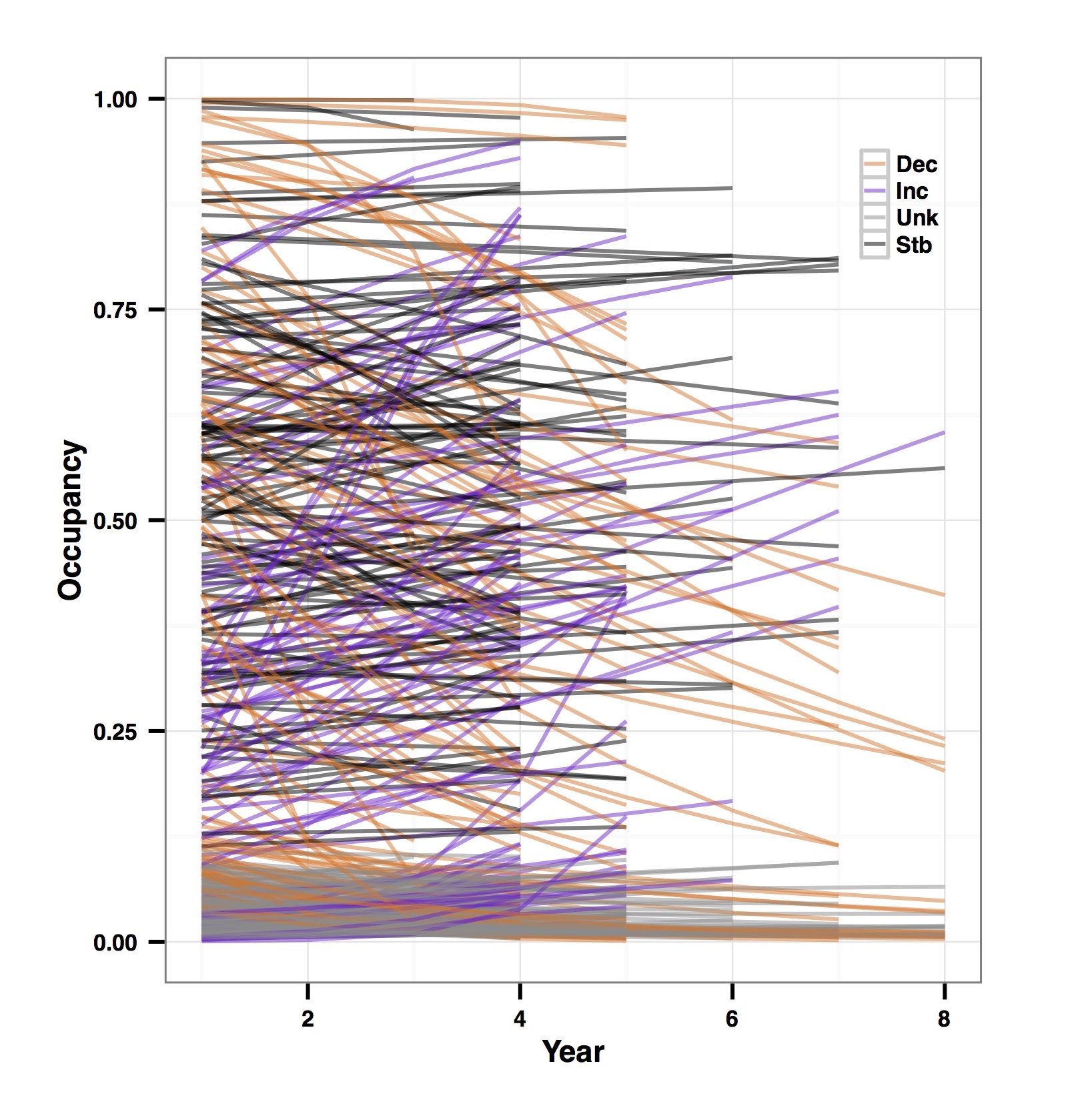

Supplement: S3 Fig — Each line depicts the trend of a particular species population monitored by TEAM. Color depicts significantly decreasing (orange), significantly increasing (purple), unknown (gray), or stable (black) occupancy trends. See S2 Table for numerical data. (TIF) [file pbio.1002357.s003.tif]

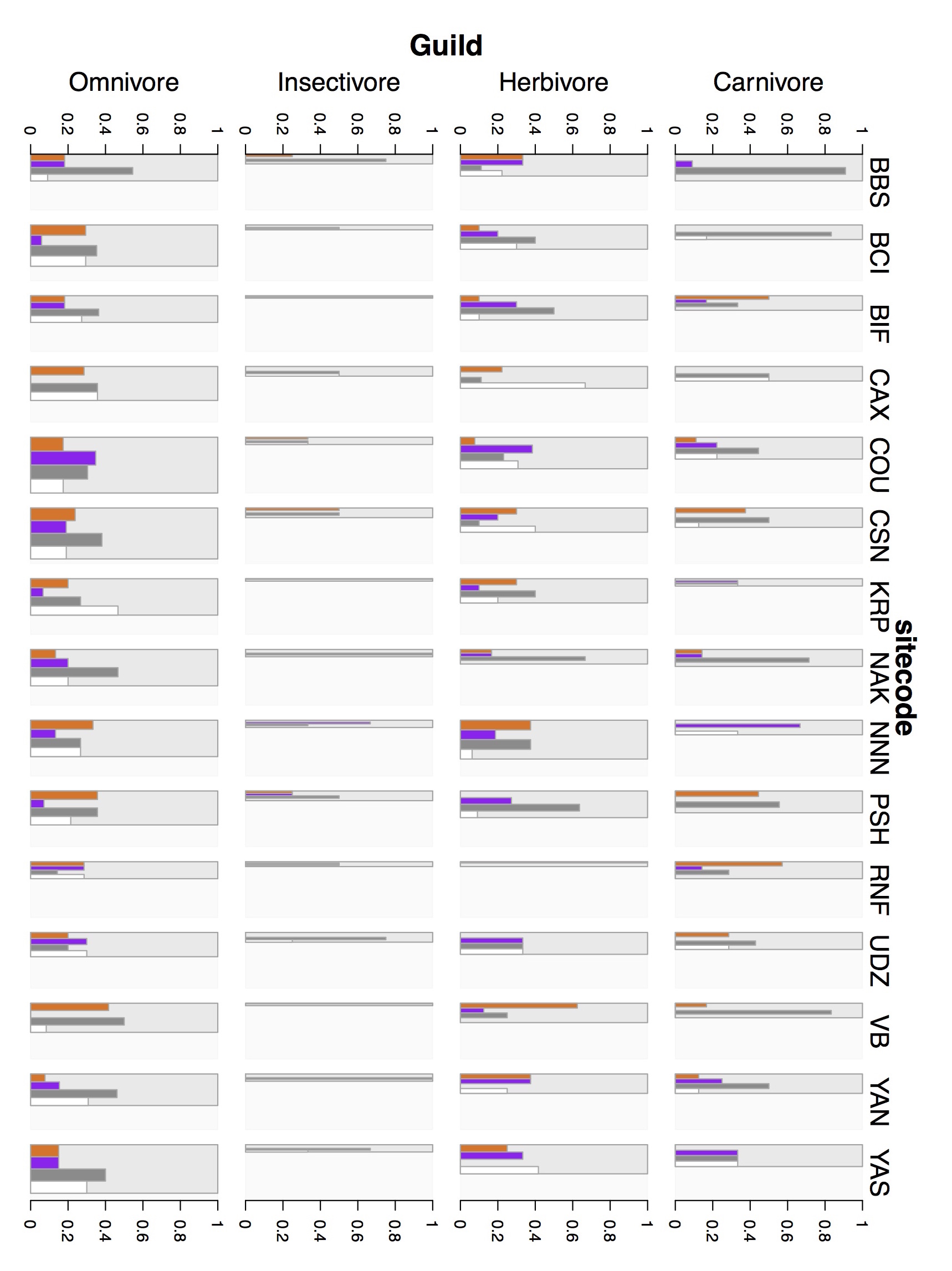

Supplement: S4 Fig — The proportion of populations with decreasing (orange), increasing (purple), unknown (gray), or stable (white) occupancy for each guild at each site; n = 511 populations. See S1 Table for site information corresponding to the three-letter site codes. Bar length illustrates the proportion of each occupancy status, and bar width illustrates the sample size for each guild at each site. See S2 Table for numerical data. (TIF) [file pbio.1002357.s004.tif]

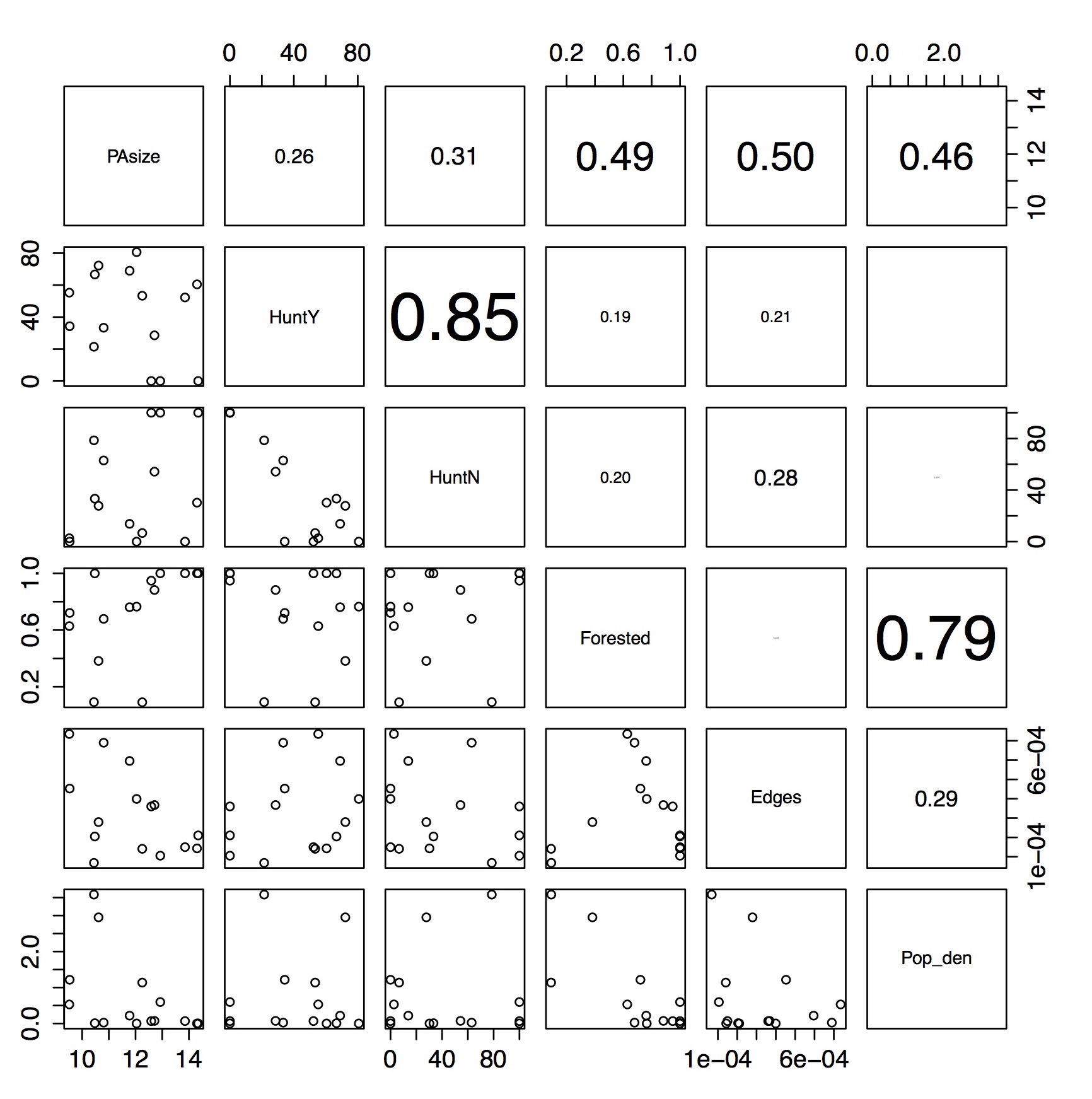

Supplement: S5 Fig — Upper triangular portion of the matrix contains Pearson correlation coefficients, with the font size proportional to the correlation coefficient. Lower triangular portions of the matrix contain pairwise scatter plots to illustrate associations. “PAsize” is the log of the size of the protected area in hectares. “HuntY” is the proportion of populations at a site that was reported as hunted. “HuntN” is the proportion of populations at a site that was reported as not hunted. “Forested” is the proportion of the ZOI that was forested. “Edges” is the edge density of the ZOI, and “Pop_den” is the human population density in the ZOI. See S1 Table for numerical data. (TIF) [file pbio.1002357.s005.tif]

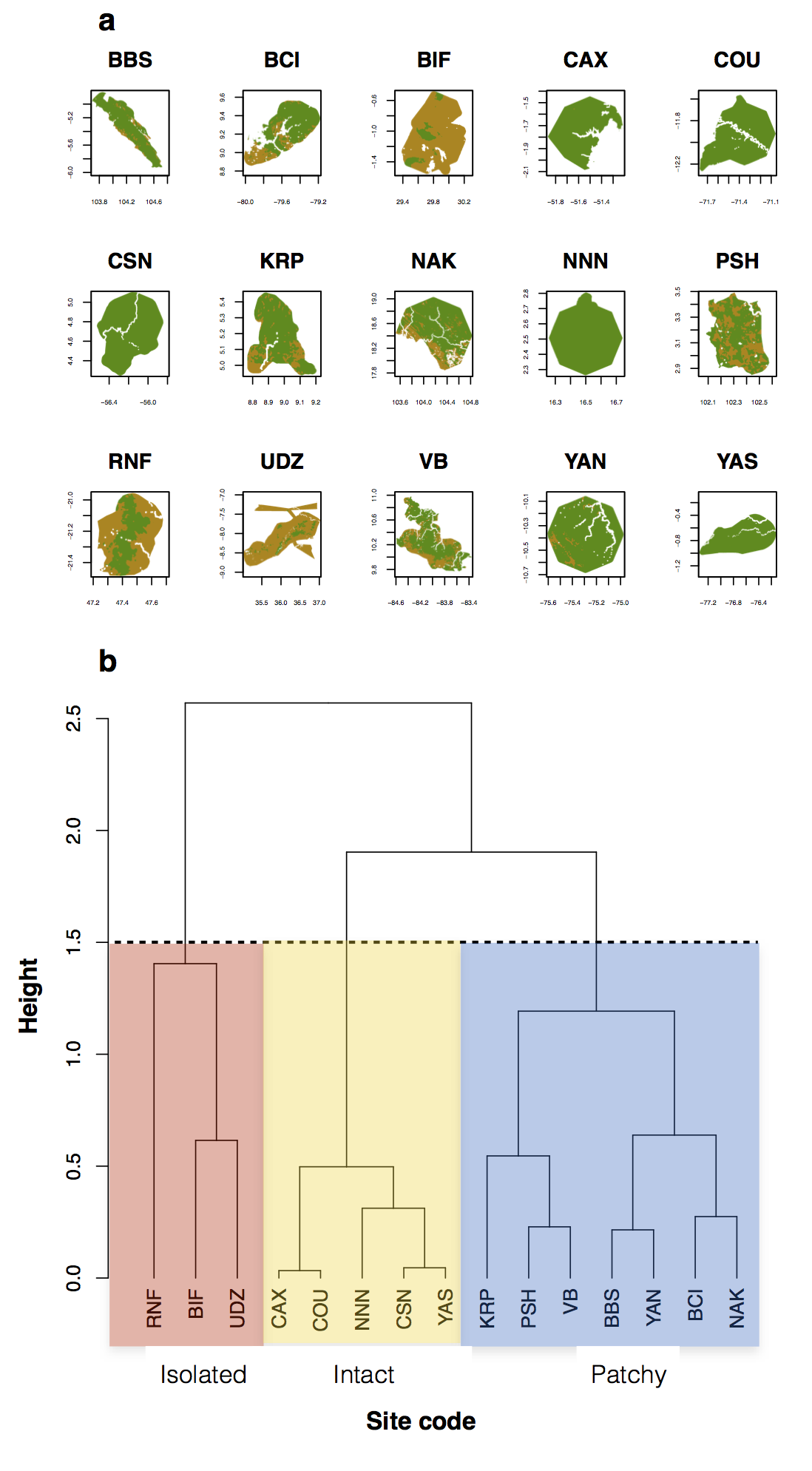

Supplement: S6 Fig — (a) Green represents forest cover and brown represents non-forested cover for the ZOI of each protected area (Materials and Methods). Bold text is the code for each TEAM site. x- and y-axis labels are degrees latitude and longitude, respectively. (b) The UPGMA cluster analysis was based on two measures of landscape connectivity: proportion of forested landscape and edge density. The cluster dendrogram depicts three clusters of similar landscapes based on a height (i.e., cluster agglomeration value) of 1.5. See S1 Table for numerical data and corresponding site information. (TIF) [file pbio.1002357.s006.tif]

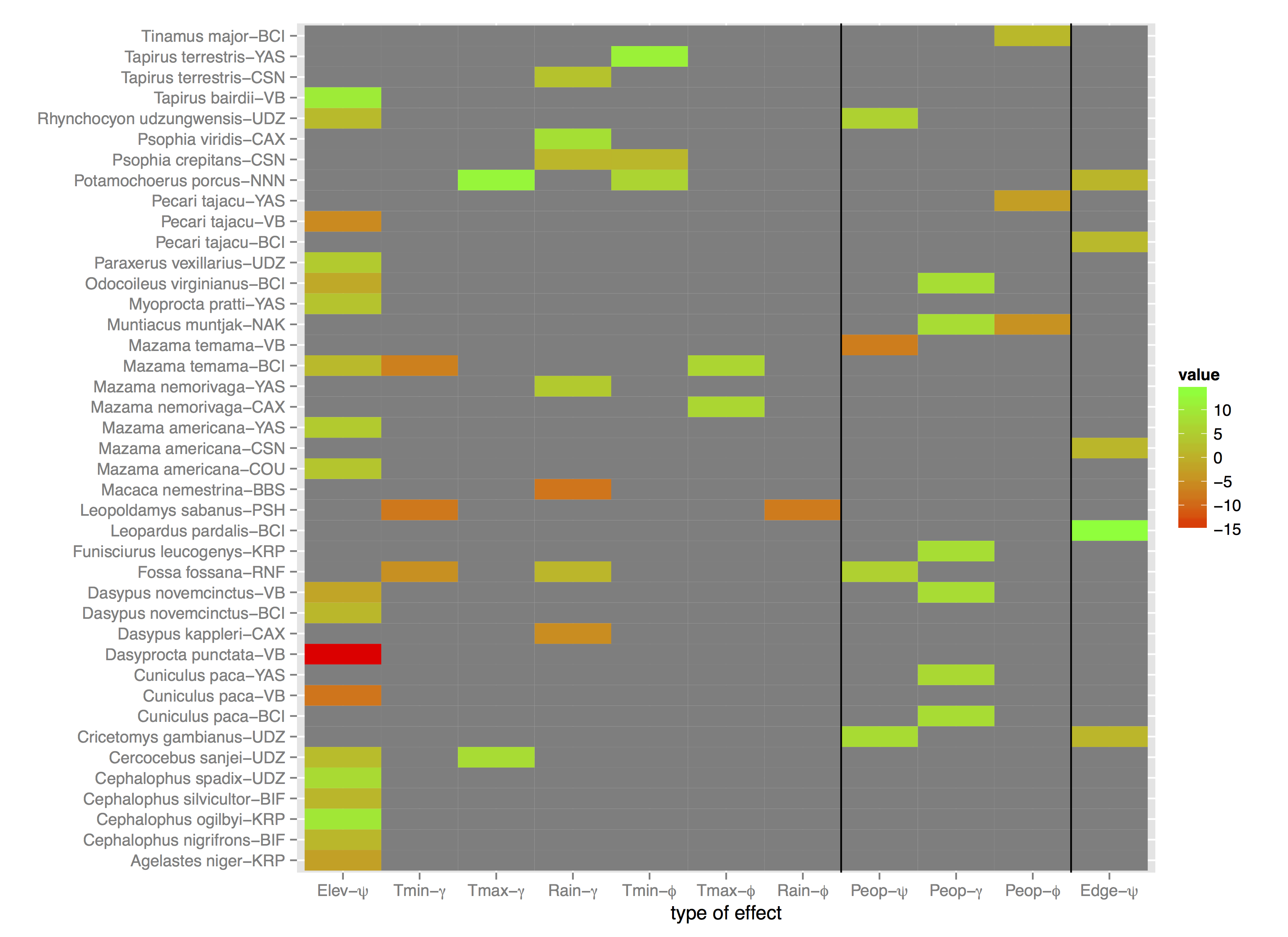

Supplement: S7 Fig — Covariates of initial occupancy (psi), colonization (lambda), and survival (phi) parameters for populations modeled as Case 1. Colors depict coefficient values of covariates identified as important using indicator variable selection (Materials and Methods). Gray shading represents covariates that were not identified as important. The y-axis labels populations by species name and site code. See S1 and S2 Tables for corresponding site and species information. See S6 Table for numerical data. (TIF) [file pbio.1002357.s007.tif]
