# Supplementary material for: Standardized Assessment of Biodiversity Trends in Tropical Forest Protected Areas: The End Is Not in Sight
Source: PLoS Biol. 2016 Jan 19;14(1):e1002357. doi: 10.1371/journal.pbio.1002357 (PMC4718630; doi:10.1371/journal.pbio.1002357)
Supplement: S1 Results — (DOCX) [file pbio.1002357.s008.docx]

**S1 Results**

*Occupancy covariates*

The populations that were modeled as Case 1 and had potentially important covariates for occupancy, extinction or colonization are shown in S7 Fig. The most common covariate was the effect of elevation on initial occupancy (N = 19), which suggests preferences for higher elevations for populations with positive (green) values and preferences for lower elevations for populations with negative (red) values. Annual minimum temperature was an important predictor of colonization for only three populations and of survival for five populations. Annual temperature maximum was an important predictor of colonization for six populations, but survival for only one population. Four populations exhibited important effects of rainfall on colonization and two others on survival. The presence of humans was an important covariate of initial occupancy for three populations, colonization for three populations and survival for seven populations. Few populations exhibited positive responses to the presence of humans, even though humans potentially included tourists and park patrol guards in addition to poachers. Lastly, distance to the nearest edge had important effects on the initial occupancy of two populations (S7 Fig).

*Population occupancy status analysis (α = 0.008)*

Class*:* The proportion of populations with decreasing, increasing, stable or unknown occupancy did not differ significantly between birds and mammals (G-test, G = 4.89, df = 3, p = 0.18, N = 511 populations) (S1a Fig).

IUCN status*:* The proportion of populations with decreasing, increasing, stable or unknown occupancy did not differ significantly by IUCN status (Data Deficient, Least Concern, Near Threatened, Threatened [53]) (G-test, G = 8.94, df = 9, p = 0.44) (S1b Fig).

Body size and guild*:* The proportion of populations with decreasing, increasing, stable or unknown occupancy did not differ significantly by body mass categories (G-test, G = 15.77, df = 6, p = 0.02, N = 511, S1c Fig). Among guilds, there were no significant differences in decreasing, stable or increasing occupancy (G-test, G =3.38, df = 6, p = 0.76), but there was significant variation among guilds in the proportion of unknown occupancy (G-test, G = 25.24, df = 9, p = 0.003) (S1d Fig).

Within guilds, there were no significant differences in occupancy status based on body size categories for carnivores (G-test, G = 10.48, df = 6, p = 0.11), herbivores (G-test, G = 5 .23, df = 6, p = 0.51), or insectivores (G-test, G = 5.66, df = 6, p = 0.46), but a larger proportion of small-bodied omnivores were classified as unknown occupancy (G-test, G = 27.09, df = 6, p < 0.001).

Landscape*:* The proportion of populations with decreasing, stable, or increasing occupancy did not vary significantly among landscape types (G-test, G = 5.17, df = 4, p = 0.27), but there was significant variation in the proportion of unknown occupancy populations among landscape types (G-test, G = 18.53, df = 6, p = 0.005). There were no significant differences in the proportion of increasing, decreasing, unknown or stable occupancy populations between isolated and patchy landscapes (G-test, G = 4.58, df = 3, p = 0.21) or between intact and isolated landscapes (G-test, G = 2.08, df = 3, p = 0.56), but there were significant differences between intact and patchy landscapes (G-test, G = 17.15, df = 3, p < 0.001). Taken together, these results suggest that patchy landscapes have a significantly higher proportion of unknown occupancy populations compared to intact or isolated landscapes. N = 511 populations. (S1e Fig).

Hunting*:* Of the 511 populations, 203 were reported as hunted, 94 as unknown, and 214 as not hunted (S2 Table). The proportion of populations hunted (hereafter “hunting”) differed significantly among TEAM sites (G = 487.99, df = 28, p < 0.001). Three field managers reported that there was no hunting (COU, NNN, YAN), while there were two sites at which all species were either hunted or of unknown hunting status (NAK, YAS). Hunting differed significantly based on surrounding landscape type (G = 25.83, df = 4, p < 0.001, N = 511). Although there were no significant differences in hunting between patchy and isolated landscapes (G-test, G = 3.09, df = 2, p = 0.21, N = 511), there was significantly more hunting in patchy than intact landscapes (G-test, G = 24.32, df = 2, p < 0.001, N = 511). The proportion of populations with decreasing, stable, or increasing occupancy did not differ significantly based on hunting status (G-test, G = 6.10, df = 4, p = 0.192), but unknown occupancy populations differed significantly by hunting status (G-test, G = 25.06, df = 6, p < 0.001, N = 391), with fewer hunted populations that were of unknown occupancy status.

Continuous predictors*:* Based on AIC comparison, none of the site-level continuous variables (i.e., protected area size, human density, effective mesh size, edge density, proportion hunted, proportion not hunted, or years of camera trap data) outcompeted the null model based on the delta AIC > 2 for the proportion of increasing or for the proportion of decreasing occupancy status populations per site (S3 Table).
