# Supplementary material for: Standardized Assessment of Biodiversity Trends in Tropical Forest Protected Areas: The End Is Not in Sight
Source: PLoS Biol. 2016 Jan 19;14(1):e1002357. doi: 10.1371/journal.pbio.1002357 (PMC4718630; doi:10.1371/journal.pbio.1002357)
Supplement: S1 Table — Including the full site name, site code, country, number of years of camera trap data, number of populations monitored by TEAM, percent of populations for each occupancy status, percent of monitored populations that were reported as hunted or not hunted, landscape connectivity, camera trap sampling area, protected area size, area of the ZOI, human population density (per ha) in the ZOI, and two measures of structural connectivity in the ZOI (proportion forested and edge density). (PDF) [file pbio.1002357.s009.pdf]

Code	Site	Country	Years of data	Populations	Landscape	% Decreasing	% Increasing	% Unknown	% Stable	% Hunted	% Not Hunted	Human Density	Forested	Edge Density	CT Sampling Area (ha)	Protected Area (ha)	ZOI Area (ha)
BBS	Bukit Barisan	Indonesia	5	35	Patchy	17.14	17.14	57.14	8.57	28.6	54.3	0.073	0.883	0.0004	20811	331155	616624
BCI	Barro Colorado Nature Monument - Soberania National Park	Panama	5	35	Patchy	17.14	8.57	45.71	28.57	34.3	0	1.216	0.721	0.0005	27445	13800	431936
BIF	Bwindi Impenetrable Forest	Uganda	4	28	Isolated	21.43	21.43	42.86	14.29	21.4	78.6	3.584	0.092	0.0001	31782	34276	808077
CAX	Caxiuana	Brazil	4	33	Intact	18.18	0	33.33	48.48	66.7	33.3	0.009	0.999	0.0002	23594	35407	293966
COU	Cocha Cashu - Manu National Park	Peru	4	48	Intact	14.58	31.25	31.25	22.92	0	100	0.002	1.000	0.0002	20132	1704506	371989
CSN	Central Suriname Nature Reserve	Suriname	7	43	Intact	30.23	13.95	34.88	20.93	60.5	30.2	0.001	1.000	0.0001	22534	1630234	504910
KRP	Korup National Park	Cameroon	3	29	Patchy	24.14	10.34	31.03	34.48	69	13.8	0.219	0.761	0.0006	15423	130348	171190
NAK	Nam Kading	Laos	5	31	Patchy	12.9	16.13	61.29	9.68	80.6	0	0.158	0.765	0.0004	23098	169569	1125109
NNN	Nouabali Ndoki	Republic of Congo	5	40	Intact	27.5	27.5	27.5	17.5	0	100	0.005	1.000	0.0001	22058	411653	229665
PSH	Pasoh Forest Reserve	Malaysia	4	38	Patchy	26.32	13.16	50	10.53	55.3	2.6	0.487	0.628	0.0007	25759	13610	281307
RNF	Ranomafana	Madagascar	4	18	Isolated	44.44	22.22	22.22	11.11	72.2	27.8	0.707	0.382	0.0003	14239	40705	211114
UDZ	Udzungwa	Udzungwa	6	30	Isolated	13.33	20	36.67	30	53.3	6.7	0.321	0.091	0.0001	14673	209538	1942132
VB	Volcan Barva	Costa Rica	8	27	Patchy	44.44	3.7	48.15	3.7	33.3	63	2.589	0.679	0.0007	21049	49317	853418
YAN	Yanachaga Chimillen National Park	Peru	4	32	Patchy	15.63	21.88	40.63	21.88	0	100	0.057	0.948	0.0004	17126	293234	366834
YAS	Yasuni	Ecuador	4	44	Intact	13.64	22.73	29.55	34.09	52.3	0	0.037	1.000	0.0001	22125	1040687	659508
