# Supplementary material for: Standardized Assessment of Biodiversity Trends in Tropical Forest Protected Areas: The End Is Not in Sight
Source: PLoS Biol. 2016 Jan 19;14(1):e1002357. doi: 10.1371/journal.pbio.1002357 (PMC4718630; doi:10.1371/journal.pbio.1002357)
Supplement: S3 Table — For (a) the proportion of decreasing occupancy status populations per site and (b) the proportion of increasing occupancy status populations per site. For both decreasing and increasing occupancy status proportions, the null models with no covariates performed better (delta AIC > 2) than all other models. (PDF) [file pbio.1002357.s011.pdf]

a.	Intercept	Beta	df	logLik	AICc	delta	weight
Null Model	-1.22	NA	1	-3.87	10.05	0	0.36
% Populations Not Hunted	-1.23	0.06	2	-3.87	12.74	2.69	0.09
ZOI Edge Density	-1.23	0.18	2	-3.87	12.74	2.7	0.09
% Populations Hunted	-1.23	0.09	2	-3.87	12.74	2.7	0.09
Human Population Density	-1.23	0.2	2	-3.87	12.75	2.7	0.09
Proportion ZOI Forested	-1.23	-0.09	2	-3.87	12.75	2.7	0.09
Protected Area Size	-1.22	-0.17	2	-3.88	12.76	2.71	0.09
Years of Camera Trap Data	-1.23	0.21	2	-3.89	12.78	2.73	0.09
**b.**							
Null Model	-1.61	NA	1	-2.73	7.78	0	0.36
Proportion ZOI Forested	-1.62	-0.03	2	-2.73	10.47	2.69	0.09
Human Population Density	-1.61	-0.13	2	-2.74	10.47	2.7	0.09
Years of Camera Trap Data	-1.65	-0.19	2	-2.74	10.48	2.7	0.09
% Populations Not Hunted	-1.65	0.27	2	-2.75	10.5	2.72	0.09
% Populations Hunted	-1.64	-0.3	2	-2.75	10.51	2.73	0.09
ZOI Edge Density	-1.63	-0.36	2	-2.76	10.51	2.73	0.09
Protected Area Size	-1.61	0.35	2	-2.76	10.52	2.74	0.09
