# Supplementary material for: Standardized Assessment of Biodiversity Trends in Tropical Forest Protected Areas: The End Is Not in Sight
Source: PLoS Biol. 2016 Jan 19;14(1):e1002357. doi: 10.1371/journal.pbio.1002357 (PMC4718630; doi:10.1371/journal.pbio.1002357)
Supplement: S1 Text — (PDF) [file pbio.1002357.s015.pdf]

Case 1 Model

```
model {

  # Priors for constant parameters

  alpha ~ dnorm(0,0.01)
  alphap ~ dnorm(0,0.01)
  alphag ~ dnorm(0,0.01)

  # Priors for parameters associated with a covariate, but the parameter is time
invariant
  for(i in 1:11){
  beta[i] ~ dnorm(0,0.01)
  w[i] ~ dbern(0.5)
  }


  #Ecological Model-define gamma and phi

  for (i in 1:nsite){
   for (t in 2:nyear){
    logit(gamma[i,t-1])<- alphag + w[4]*beta[4]*rainfall6[t]
    + w[5]*beta[5]*maxtemp6[t]
    + w[6]*beta[6]*mintemp6[t]
    + w[10]*beta[10]*people[i,t]



    logit(phi[i,t-1])<-alphap + w[7]*beta[7]*rainfall6[t]
    + w[8]*beta[8]*maxtemp6[t]
    + w[9]*beta[9]*mintemp6[t]
    + w[11]*beta[11]*people[i,t]
   }
  }

  #Ecological Model - define psi1 and subsequent ones
  for (i in 1:nsite){
   logit(psi1[i])<-alpha + w[1]*beta[1]*elevation[i,1]
   + w[2]*beta[2]*people[i,1]
   + w[3]*beta[3]*edge[i,1]

   z[i,1] ~ dbern(psi1[i])

    for (t in 2:nyear){
     muZ[i,t]<- z[i,t-1]*phi[i,t-1] + (1-z[i,t-1])*gamma[i,t-1]
```

```
      z[i,t] ~ dbern(muZ[i,t])
    }#t
  } #i

 #Observation process - no covariates
 for(t in 1:nyear){
  p[t] ~ dunif(0,1)
 } #t

 for (i in 1:nsite){
  for (j in 1:nrep){
    for (t in 1:nyear){
      muy[i,j,t] <- z[i,t]*p[t]
      y[i,j,t] ~ dbern(muy[i,j,t])
    } #t
  } #j
 } #i

 # Derived quantities (finite sample estimates)

 for(t in 1:nyear){
  psi[t]<-sum(z[1:nsite,t])/nsite
 }#t

}
```

Case 2 Model

```
model {

# Specify priors
psi1 ~ dunif(0, 1) #first year occupancy

phi ~ dunif(0, 1) # apparent survival
gamma ~ dunif(0, 1) # colonization
p ~ dunif(0, 1) # detection probability

# Ecological submodel: Define state conditional on parameters
for (i in 1:nsite){
  z[i,1] ~ dbern(psi1)
  for (k in 2:nyear){
    muZ[i,k]<- z[i,k-1]*phi + (1-z[i,k-1])*gamma
    z[i,k] ~ dbern(muZ[i,k])
    } #k
  } #i

# Observation model
for (i in 1:nsite){
  for (j in 1:nrep){
    for (k in 1:nyear){
      muy[i,j,k] <- z[i,k]*p
      y[i,j,k] ~ dbern(muy[i,j,k])
      } #k
    } #j
  } #i


# Derived parameters: Sample occupancy

for (k in 1:nyear){
  psi[k]<-sum(z[1:nsite,k])/nsite
  }
}
```

Case 3 Code

```
require(binom)


#Function that creates some fake data for a species
#that is rare
f.data.generator<-function(sites,days,psi,p,phi,gamma,nyears) {
 #first year of data
 y1<-matrix(NA,nr=sites,nc=days)
 #generate the expected occupancies
 z<-rbinom(sites,1,psi)
 #generate the observations
 for(i in 1:sites)
  y1[i,]<-rbinom(days,1,z[i]*p)
 #subsequent years
 #three dimensional matrix to store the results
 yk<-array(NA,dim=c(sites,days,nyears))
 yk[,,1]<-y1
 for(k in 2:nyears){
  #generate the deterministic part of the model
  occ<-apply(yk[,,k-1],1,max,na.rm=T)
  z<-rbinom(sites,1,occ*phi+(1-occ)*gamma)
  #generate the observations
  for(i in 1:sites)
   yk[i,,k]<-rbinom(days,1,z[i]*p)

 }
 yk
}

#function to calculate the mode of a distribution
f.mode<-function(data){
 qwe<-density(data)
 qwe$x[which(qwe$y==max(qwe$y))]

}

# Create a new data set



data<-
f.data.generator(sites=60,days=15,psi=0.05,p=.05,phi=0.4,gamma=0.1,nyears=5)
#simulating some holes in the data
data[31:60,1:7,]<-NA
```

```
data[1:30,8:15,]<-NA
data[60,,]<-NA
data[13,,]<-NA

nyears <- dim(data)[3]
# calculate whether the species was present or absent at each point each year
pa.year<-apply(data,c(1,3),max,na.rm=T)
pa.year[pa.year=="-Inf"]<-NA

# how many camera traps were functional?
n.cams <- apply(pa.year,2,function(x) {sum(!is.na(x))})

# How many detections of each species per year?
n.dets <- apply(pa.year,2,sum,na.rm=T)

# Average number of detections? If mean.dets < 5 we use this process to calculate
# occupancy
mean.dets <- mean(n.dets)

# naive occupancy
occ <- n.dets/n.cams
#new.occ will contain 1000 realizations of each
new.occ <- matrix(NA,nr=1000,nc=nyears)

for(i in 1:nyears){
  conf.bin <- binom.bayes(x=n.dets[i],n=n.cams[i],conf.level=0.95,type="highest")
  mod.bin <- binom.bayes.densityplot(bayes=conf.bin)
  new.occ[,i] <-
sample(x=mod.bin$data$xx,size=1000,replace=T,prob=mod.bin$data$yy)
}

# Check everything makes sense and graph it
conf.lims <- apply(new.occ,2,quantile,c(0.025,0.975))
occ.mode <- apply(new.occ,2,f.mode)

#plot mode of the distribution plus confidence limits
plot(1:nyears,occ.mode,t='l',ylim=c(0,1))
lines(1:nyears,conf.lims[1,],lty=2)
lines(1:nyears,conf.lims[2,],lty=2)
#observed
points(1:nyears,occ)
```

R Code

```
#### Beaudrot, Ahumada, et al. PLoS Biology
#### Examine TEAM population status based on WPI output

rm(list=ls())
library(Deducer)
library(extracat)
library(MuMIn)
library(ggplot2)
library(ggthemes)

####################  Data input and formatting
######################
SiteData <- read.csv(file="SiteData_TableS1.csv")
PopData <- read.csv(file="PopulationData_TableS2.csv")
    Merged <- merge(SiteData, PopData, by.x="Code", by.y="Site")
      mass_cat <- ifelse(Merged$mass<1000, "1", ifelse(Merged$mass>=1000 &
Merged$mass<10000, "2", ifelse(Merged$mass>=10000, "3", NA)))
        lab <- ifelse(mass_cat=="1", "0.1 - 1 kg",
             ifelse(mass_cat=="2", "1 - 10 kg",
               ifelse(mass_cat=="3", "10 kg or more", NA)))

# Overall data for analysis
WPI.all <- data.frame("Mass"=lab, Merged)
  WPI.all$Case <- factor(WPI.all$Case)

# Excludes Unknown Population Status
WPI <- WPI.all[WPI.all$Status!="Unknown",]
  WPI$Status <- factor(WPI$Status)

# Excludes sites with no reported hunting (NNN, YAN and COU)
HuntData <- WPI.all[WPI.all$Code!="NNN" & WPI.all$Code!="COU" &
WPI.all$Code!="YAN",]

# Data frame with information on the 244 species
unique.species <- WPI.all[unique(WPI.all$Species),c(1, 20:21, 23:25)]

##################### END DATA FORMATTING
######################



##################### BEGIN SUMMARIZATION ###############

# Determine # of unique species
```

```
length(unique(WPI.all$Species))
length(unique(WPI.all$Species[WPI.all$Class=="Aves"]))
length(unique(WPI.all$Species[WPI.all$Class=="Mammalia"]))

# Determine % of populations for each population status
table(WPI.all$Status)
(table(WPI.all$Status)/dim(WPI.all)[1])*100

# Determine breakdown and % populations by IUCN categories
table(WPI.all$IUCN)
(table(WPI.all$IUCN)/dim(WPI.all)[1])*100

# Determine # species per IUCN category
table(unique.species$IUCN)

# Determine breakdown and % by Class
table(WPI.all$Class)
table(WPI.all$Status, WPI.all$Class)
(table(WPI.all$Status, WPI.all$Class)[,1]/table(WPI.all$Class)[1])*100
(table(WPI.all$Status, WPI.all$Class)[,2]/table(WPI.all$Class)[2])*100

# Determine the # of populations evaluated and not evaluated (i.e. unknown)
sum(table(WPI.all$Status, WPI.all$Class)[4,]) # total unknown populations
table(WPI.all$Status, WPI.all$Class)[4,] # unknown populations by class
sum(table(WPI.all$Status, WPI.all$Class)[-4,]) # total evaluted populations
colSums(table(WPI.all$Status, WPI.all$Class)[-4,]) # evaluated populations by class

# Determine the # of unique species not evaluated (i.e. unknown)
length(unique(WPI.all$Species[WPI.all$Status=="Unknown"])) # Overall
length(unique(WPI.all$Species[WPI.all$Status=="Unknown" &
WPI.all$Class=="Aves"])) # Birds
length(unique(WPI.all$Species[WPI.all$Status=="Unknown" &
WPI.all$Class=="Mammalia"])) # Mammals

# Determine the # of unique species evaluated (i.e. not unknown)
length(unique(WPI.all$Species[WPI.all$Status!="Unknown"])) # Overall
length(unique(WPI.all$Species[WPI.all$Status!="Unknown" &
WPI.all$Class=="Aves"])) # Birds
length(unique(WPI.all$Species[WPI.all$Status!="Unknown" &
WPI.all$Class=="Mammalia"])) # Mammals

# Determine the number of species with only unknown populations observed at a
single site only
SingleSite <- table(WPI.all$Species, WPI.all$Status)
SingleSite <- SingleSite[rowSums(SingleSite)==1,]
SingleSite <- SingleSite[,4]
```

```
length(SingleSite[SingleSite>0]) # Number of unknown populations at only one site
SinglePops <- names(SingleSite[SingleSite>0]) # Population names
SinglePops <- WPI.all[match(SinglePops, WPI.all$Species),]
table(SinglePops$Class) # By Class
table(SinglePops$IUCN) # By IUCN status

# Determine the number of species with only unknown populations at multiple sites
# Extract species with multiple populations
PopsPerSpecies <- table(WPI.all$Species,WPI.all$Status)
PopsPerSpecies <- cbind(PopsPerSpecies, tot=rowSums(PopsPerSpecies))
PopsPerSpecies <- as.data.frame(PopsPerSpecies)
PopsPerSpecies <- PopsPerSpecies[PopsPerSpecies$tot>1,] # Species at multiple
sites
dim(PopsPerSpecies) # Number of species (any population status) at multiple sites
UnknownOnly <-
PopsPerSpecies[PopsPerSpecies$tot==PopsPerSpecies$Unknown,]
UnknownOnly <- match(as.character(WPI.all$Species), rownames(UnknownOnly))
UnknownOnly <- cbind(WPI.all, UnknownOnly)
UnknownOnly <- na.omit(UnknownOnly)
length(unique(UnknownOnly$Species)) # Number of species with unknown only
populations at multiple sites
dim(UnknownOnly) # Number of populations for species with unknown only
populations at multiple sites
# Examine the attributes of the consistently unknown species
UnknownSpecies <- UnknownOnly[duplicated(UnknownOnly$Species)==FALSE,]
table(UnknownSpecies$IUCN)
# Determine the number of unknown populations from species evaluated at other
TEAM sites
table(WPI.all$Status)[4] - dim(SinglePops)[1] - dim(UnknownOnly)[1]
# Examine the number of populations of the consistently unknown species
sort(table(UnknownOnly$Species))

# Identify number of populations with unknowns excluded
length(unique(WPI$Species))
length(unique(WPI$Species[WPI$Class=="Aves"]))
length(unique(WPI$Species[WPI$Class=="Mammalia"]))


# Summarize species body size and guild data
summary(unique.species$mass)
table(unique.species$Mass, unique.species$Class)
table(unique.species$Guild, unique.species$Class)


# Summarize populations by hunting status
table(WPI.all$Hunted)
```

```
############### END DATA SUMMARIZATION ####################


############### BEGIN GOODNESS OF FIT TESTS ###############

# Create matrix for G test across Case types
table(WPI.all$Case, WPI.all$Status) # Simplify table to exclude zero values
    mdat <- matrix(c(31, 32, 48,  26, 33, 29,  48, 63, 201), nrow=3, ncol=3,
byrow=FALSE,
          dimnames=list(c("Case1", "Case2", "Case3"),
                   c("Decreasing", "Increasing", "Stable")))

# Test proportion of unknown/dec/stable/inc across model Case types
likelihood.test(mdat) # All populations
likelihood.test(mdat[-3,]) # Exclude Case3 (populations with < 5 observations per
year)

# Test proportions across other attributes with (WPI.all) and without (WPI)
unknown populations

# Population status by class
likelihood.test(table(WPI$Class, WPI$Status))
likelihood.test(table(WPI.all$Class, WPI.all$Status))

# Population status by IUCN status
likelihood.test(table(WPI$IUCN, WPI$Status))
likelihood.test(table(WPI.all$IUCN, WPI.all$Status))

# Population status by body size category
likelihood.test(table(WPI$Mass, WPI$Status))
likelihood.test(table(WPI.all$Mass, WPI.all$Status))

# Population status by guild
likelihood.test(table(WPI$Guild, WPI$Status))
likelihood.test(table(WPI.all$Guild, WPI.all$Status))

# Population status within guilds by body size
likelihood.test(table(WPI.all$Status, WPI.all$Mass, WPI.all$Guild)[,,1])
likelihood.test(table(WPI.all$Status, WPI.all$Mass, WPI.all$Guild)[,,2])
likelihood.test(table(WPI.all$Status, WPI.all$Mass, WPI.all$Guild)[,,3])
likelihood.test(table(WPI.all$Status, WPI.all$Mass, WPI.all$Guild)[,,4])

# Population status by landscape type
likelihood.test(table(WPI$Landscape, WPI$Status))
likelihood.test(table(WPI.all$Landscape, WPI.all$Status))
```

```
likelihood.test(table(WPI.all$Landscape, WPI.all$Status)[-1,])
likelihood.test(table(WPI.all$Landscape, WPI.all$Status)[-2,])
likelihood.test(table(WPI.all$Landscape, WPI.all$Status)[-3,])

# Hunting all sites
likelihood.test(table(WPI.all$Hunted, WPI.all$Code))
likelihood.test(table(WPI.all$Hunted, WPI.all$Class))
likelihood.test(table(WPI.all$Hunted, WPI.all$Landscape))
likelihood.test(table(WPI.all$Hunted, WPI.all$Landscape)[,-1])
likelihood.test(table(WPI.all$Hunted, WPI.all$Landscape)[,-2])

# Hunting only at 12 sites with hunted populations reported
likelihood.test(table(HuntData$Hunted, HuntData$Class))
likelihood.test(table(HuntData$Hunted, HuntData$IUCN))
likelihood.test(table(HuntData$Hunted, HuntData$Guild))
likelihood.test(table(HuntData$Hunted, HuntData$Mass))
likelihood.test(table(HuntData$Hunted, HuntData$Landscape))
likelihood.test(table(HuntData$Hunted, HuntData$Status))
likelihood.test(table(HuntData$Hunted, HuntData$Status)[,-4])

# Population status by hunting status
likelihood.test(table(WPI$Hunted, WPI$Status))
likelihood.test(table(WPI.all$Hunted, WPI.all$Status))



############## END GOODNESS OF FIT TESTS #####################


############## BEGIN DATA VIZUALIZATION ######################

# Distribution of population trends and status classifications
WPIplot1 <- ggplot(WPI.all, aes(Slope, fill=factor(Status))) +
  geom_histogram(aes(y=..count..), binwidth=0.05) +
  xlim(-1.5, 1.5) +
  xlab("Median occupancy trend coefficient") +
  ylab("Populations")

WPIplot1 + theme_bw() + theme(panel.grid.major = element_blank(),
panel.grid.minor = element_blank()) +
  theme(legend.position = c(0.15, 0.77)) +
  scale_fill_manual(values=c( "chocolate2", "purple3", "gray55","cornsilk2"),
name="Population Status")


# Plot proportions by multiple categorical variables
```

```
pdf(file="WPI_Extracat_SupplementalFigures.pdf")

rmb(formula= ~  Class + Status, data=WPI.all, col=c("chocolate2", "purple", "white",
"gray55"),
   label.opt=list(yaxis=TRUE, boxes=FALSE, lab.tv=FALSE, lab.cex=1,
varnames=TRUE), gap.prop=0.07)

rmb(formula= ~  IUCN + Status, data=WPI.all, col=c("chocolate2", "purple", "white",
"gray55"),
   label.opt=list(yaxis=TRUE, boxes=FALSE, lab.tv=FALSE, lab.cex=1,
varnames=TRUE), gap.prop=0.01)

rmb(formula= ~  Mass + Status, data=WPI.all, col=c("chocolate2", "purple", "white",
"gray55"),
   label.opt=list(yaxis=TRUE, boxes=FALSE, lab.tv=FALSE, lab.cex=1,
varnames=TRUE), gap.prop=0.01)

rmb(formula= ~  Guild + Status, data=WPI.all, col=c("chocolate2", "purple", "white",
"gray55"),
   label.opt=list(yaxis=TRUE, boxes=FALSE, lab.tv=FALSE, lab.cex=1,
varnames=TRUE), gap.prop=0.01)

rmb(formula= ~  Mass + Guild + Status, data=WPI.all, col=c("chocolate2", "purple",
"white", "gray55"),
   label.opt=list(yaxis=TRUE, boxes=FALSE, lab.tv=FALSE, lab.cex=0.7,
varnames=TRUE), gap.prop=0.1)

rmb(formula= ~ Landscape + Status, data=WPI.all, col=c("chocolate2", "purple",
"white", "gray55"),
   label.opt=list(yaxis=TRUE, boxes=FALSE, lab.tv=FALSE, lab.cex=1,
varnames=TRUE), gap.prop=0.01)

rmb(formula= ~ Landscape + Hunted, data=WPI.all, col=c("green4", "gray65",
"blue3"),
   label.opt=list(yaxis=TRUE, boxes=FALSE, lab.tv=FALSE, lab.cex=1,
varnames=TRUE), gap.prop=0.01)

rmb(formula= ~  Mass + Guild + Hunted, data=HuntData, col=c("green4", "gray65",
"blue3"),
   label.opt=list(yaxis=TRUE, boxes=FALSE, lab.tv=FALSE, lab.cex=0.7,
varnames=TRUE), gap.prop=0.07)

rmb(formula= ~ Hunted + Status, data=HuntData, col=c("chocolate2", "purple",
"white", "gray55"),
   label.opt=list(yaxis=TRUE, boxes=FALSE, lab.tv=FALSE, lab.cex=1,
varnames=TRUE), gap.prop=0.07)
```

```
rmb(formula= ~ Landscape + Guild + Status, data=WPI.all, col=c("chocolate2",
"purple", "white", "gray55"),
   label.opt=list(yaxis=TRUE, boxes=FALSE, lab.tv=FALSE, lab.cex=0.7,
varnames=TRUE), gap.prop=0.07)

rmb(formula= ~ Code + Guild + Status, data=WPI.all, col=c("chocolate2", "purple",
"white", "gray55"),
   label.opt=list(yaxis=TRUE, boxes=FALSE, lab.tv=FALSE, lab.cex=0.7,
varnames=TRUE), gap.prop=0.07)
dev.off()

############## END DATA VIZUALIZATION #####################



########BEGIN LOGISTIC REGRESSION WITH CONTINUOUS VARIABLES ###
# Examine site-level % inc and % dec as a function of site-level variables
SiteData

Dec <- cbind(SiteData$Decrease.Per, 100-SiteData$Decrease.Per)/100
m0 <- glm(Dec ~ 1, family="binomial")
m1 <- glm(Dec ~ scale(log(SiteData$PA_Area)), family="binomial")
m2 <- glm(Dec ~ scale(SiteData$PopDen), family="binomial")
m3 <- glm(Dec ~ scale(SiteData$Forested), family="binomial")
m4 <- glm(Dec ~ scale(SiteData$EdgeDensity), family="binomial")
m5 <- glm(Dec ~ scale(SiteData$Per.HuntedY), family="binomial")
m6 <- glm(Dec ~ scale(SiteData$Per.HuntedN), family="binomial")
m7 <- glm(Dec ~ scale(SiteData$CTyears), family="binomial")
Dec.table <- model.sel(m0, m1, m2, m3, m4, m5, m6, m7)

Inc <- cbind(SiteData$Increase.Per, 100-SiteData$Increase.Per)/100
m10 <- glm(Inc ~ 1, family="binomial")
m11 <- glm(Inc ~ scale(log(SiteData$PA_Area)), family="binomial")
m12 <- glm(Inc ~ scale(SiteData$PopDen), family="binomial")
m13 <- glm(Inc ~ scale(SiteData$Forested), family="binomial")
m14 <- glm(Inc ~ scale(SiteData$EdgeDensity), family="binomial")
m15 <- glm(Inc ~ scale(SiteData$Per.HuntedY), family="binomial")
m16 <- glm(Inc ~ scale(SiteData$Per.HuntedN), family="binomial")
m17 <- glm(Inc ~ scale(SiteData$CTyears), family="binomial")
Inc.table <- model.sel(m10, m11, m12, m13, m14, m15, m16, m17)
############# END LOGISTIC REGRESSION #####################
```
